# Data mining of human plasma proteins generates a multitude of highly predictive aging clocks that reflect different aspects of aging

**DOI:** 10.1111/acel.13256

**Published:** 2020-10-08

**Authors:** Benoit Lehallier, Maxim N. Shokhirev, Tony Wyss‐Coray, Adiv A. Johnson

**Affiliations:** ^1^ Department of Neurology and Neurological Sciences Stanford University Stanford California USA; ^2^ Wu Tsai Neurosciences Institute Stanford University Stanford California USA; ^3^ Paul F. Glenn Center for the Biology of Aging Stanford University Stanford California USA; ^4^ Razavi Newman Integrative Genomics and Bioinformatics Core The Salk Institute for Biological Studies La Jolla California USA; ^5^ Department of Veterans Affairs VA Palo Alto Health Care System Palo Alto California USA; ^6^ Tucson Arizona USA

**Keywords:** age‐related disease, aging, aging clock, health span, life span, longevity

## Abstract

We previously identified 529 proteins that had been reported by multiple different studies to change their expression level with age in human plasma. In the present study, we measured the q‐value and age coefficient of these proteins in a plasma proteomic dataset derived from 4263 individuals. A bioinformatics enrichment analysis of proteins that significantly trend toward increased expression with age strongly implicated diverse inflammatory processes. A literature search revealed that at least 64 of these 529 proteins are capable of regulating life span in an animal model. Nine of these proteins (AKT2, GDF11, GDF15, GHR, NAMPT, PAPPA, PLAU, PTEN, and SHC1) significantly extend life span when manipulated in mice or fish. By performing machine‐learning modeling in a plasma proteomic dataset derived from 3301 individuals, we discover an ultra‐predictive aging clock comprised of 491 protein entries. The Pearson correlation for this clock was 0.98 in the learning set and 0.96 in the test set while the median absolute error was 1.84 years in the learning set and 2.44 years in the test set. Using this clock, we demonstrate that aerobic‐exercised trained individuals have a younger predicted age than physically sedentary subjects. By testing clocks associated with 1565 different Reactome pathways, we also show that proteins associated with signal transduction or the immune system are especially capable of predicting human age. We additionally generate a multitude of age predictors that reflect different aspects of aging. For example, a clock comprised of proteins that regulate life span in animal models accurately predicts age.

## Graphical abstract

Machine learning analyses of proteins that change their expression level with age in human plasma discovered an ultra‐predictive proteomic aging clock and also unveiled widely accessible clocks that reflect different aspects of aging. For example, proteins that impact lifespan in animal models when manipulated can accurately predict age in a large human cohort comprised of 3301 individuals (aged 18–76 years).

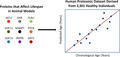

## 1 INTRODUCTION

A panel of molecules capable of predicting chronological age when modeled is referred to as an aging clock (Galkin et al., 2020a). Existing examples of human aging clocks include those comprised of methylated DNA (Hannum et al., 2013; Horvath, 2013), RNA (Mamoshina et al., 2018), proteins (Johnson et al., 2020), metabolites (Rist et al., 2017; Robinson et al., 2020), biochemical markers (Putin et al., 2016; Sagers et al., 2020), or microbiota (Galkin, et al., 2020b). For a more detailed discussion of different types of aging clocks, we recommend a comprehensive review by Galkin et al. (2020a). A recent proteomic aging clock found that individuals with a lower predicted age than their chronological age performed better on cognitive and physical tests (Lehallier et al., 2019). An RNA clock demonstrated that the difference between predicted and actual age was associated with body mass index, blood pressure, fasting glucose, and cholesterol levels (Peters et al., 2015). A much larger body of work using DNA methylation clocks has shown that patients with age‐related disease often have a higher predicted age than their chronological age (Horvath & Raj, 2018). These data suggest that aging clocks have the ability to measure biological age, which can be conceptualized as a composite measure that correlates with various health outcomes.

Given that it is not realistic to perform life span studies in humans, a prominent appeal of aging clocks is their potential ability to accelerate anti‐aging clinical trials (Horvath & Raj, 2018). Prior to and after testing of an anti‐aging intervention, biological age could be measured in a patient cohort. Theoretically, a therapy that successfully combats aging would be one where biological age is reduced compared to controls at the end of the treatment period. Repeated measurements of biological age also have the potential to be highly informative on an individual level. They could, for example, suggest whether or not someone ought to more aggressively pursue health‐promoting interventions to slow down their rate of aging. The requirement for repeat sampling necessitates a sample type that can be measured safely and easily, such as blood or saliva. Since the aging clock field is nascent, much work remains to be done to confidently determine if these theoretical applications are feasible.

In addition to existing drugs whose promising anti‐aging potential should be safely tested in humans (Partridge et al., 2020), designing novel therapies capable of improving human health span will require well‐considered molecular targets. A wide variety of approaches have been historically utilized to identify aging‐relevant targets and therapeutics, including RNAi screening in worms (Hansen et al., 2005), computational screening of the protein–drug interactome (Fuentealba et al., 2019), and omics‐level expression screening in mice (Villeda et al., 2011). As an example of the latter, young mice exposed to the blood of old mice via heterochronic parabiosis exhibit decreased synaptic plasticity as well as impairments in memory and learning. A proteomics expression screen identified that the chemokine Ccl11 was the most significantly altered protein in these heterochronic parabionts. Subsequently, treating young mice with Ccl11 was found to induce various deleterious effects in the brain (Villeda et al., 2011).

With the ultimate objective of improving human health span in mind, we sought to better understand proteomic aging clocks and to identify high‐quality protein targets that exhibit anti‐aging clinical potential. Since systematic factors are powerful regulators of aging (Pluvinage & Wyss‐Coray, 2020), we aimed to achieve these goals by comprehensively data mining human plasma proteins.

## 2 RESULTS

### 2.1 Analysis of all 529 common plasma aging proteins in a large proteomics dataset

**Figure 1:**
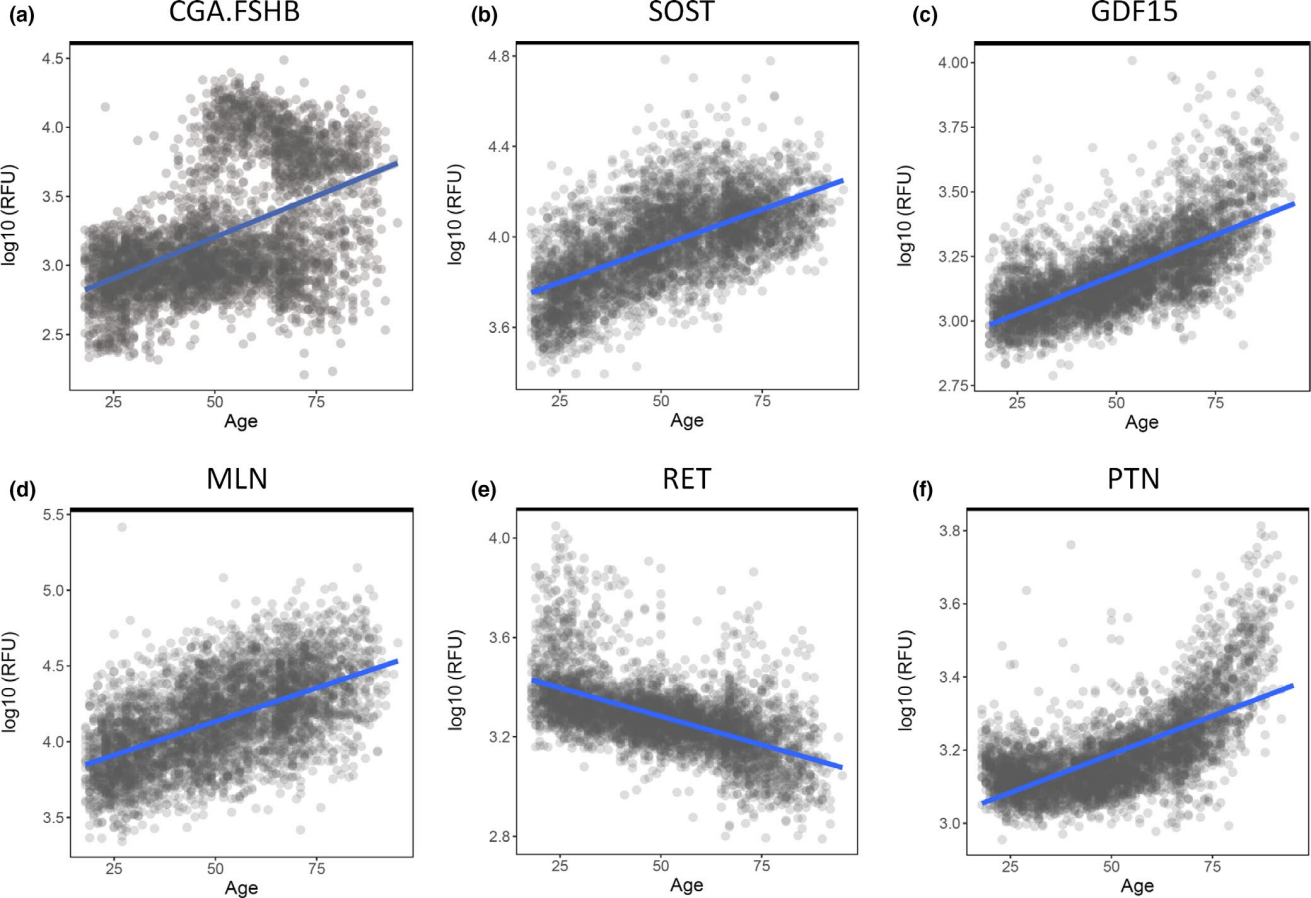
529 proteins that were previously identified to change their expression level with age in human plasma were analyzed in a large, proteomic dataset derived from 4263 healthy individuals with an age range of 18–95 years. The six proteins that exhibited the most significant change in plasma expression level with age were CGA.FSHB (a), SOST (b), GDF15 (c), MLN (d), RET (e), and PTN (f). The expression trend over time is visually shown for each protein. RFU = relative fluorescent unit

Our recent systematic review identified 529 proteins that were reported to change their expression level with age in human plasma by two or more different studies (Johnson et al., 2020). In the present study, we began analyzing these proteins by measuring their q‐value and age coefficient in a plasma proteomic dataset derived from 4263 healthy individuals with an age range of 18–95 years. Proteomic measurements were previously performed using the SOMAscan assay, which utilizes individual SOMAmers to measure different proteins (Lehallier et al., 2019). Our 529 proteins (Table S1) were condensed into 523 protein entries (Table S2) in this dataset due to some measurements containing multiple different proteins. For example, the heterotrimeric enzyme AMPK was measured using the single SOMAmer “PRKAA1.PRKAB1.PRKAG1.” Twenty‐seven proteins were not available for measurement and, of the 496 protein measurements, 476 (95.97%) significantly (*q* < 0.05) changed their expression level with age. Of these 476 significant protein entries, 115 (24.16%) trended toward a decreased expression level with age while 361 (75.84%) trended toward an increased expression level with age. These and other statistics are summarized in Table S3. The six protein measurements with the lowest *q*‐values are shown in Figure 1 and are as follows: CGA.FSHB, SOST, GDF15, MLN, RET, and PTN.

### 2.2 Many common aging plasma proteins have highly intriguing links to aging and/or health

We next looked up each common aging plasma protein in the Human Ageing Genomic Resources (HAGR) database (Tacutu et al., 2018) and found that 103 (19.47% of all 529 proteins) had a HAGR listing. After performing a comprehensive literature search, we were also able to find a tangible connection to aging and/or health for all 523 protein entries (Table S2).

Many of the connections we identified are highly intriguing. For example, injecting B2M into young mice impairs neurogenesis and cognitive function (Smith et al., 2015) while treating aged mice with Timp2 enhances cognition and synaptic plasticity (Castellano et al., 2017). Ablating *Cdon* in satellite cells hinders muscle regeneration in mice (Bae et al., 2020), mice lacking *Il6* exhibit impaired liver regeneration (Cressman et al., 1996), and the myeloid cell‐specific ablation of *Plxnb2* in mice impairs motor recovery following spinal cord injury (Zhou et al., 2020). Cardiac hypoplasia is caused by the deletion of *tmem87b* in zebrafish (Russell et al., 2014) while mice overexpressing *Nab1* are resistant to cardiac hypertrophy (Buitrago et al., 2005). Diabetes in mouse models of insulin resistance, insulin deficiency, and obesity can be reversed by the overexpression of *Igfbp2* (Hedbacker et al., 2010) and, in contrast, mice harboring a mutation in *Lep* become obese and diabetic (Zhang et al., 1994). More broadly, connections pertinent to age‐related disease, the canonical insulin/IGF1, AMPK, and TOR aging pathways (Singh et al., 2019), and lipid metabolic pathways that directly regulate aging (Johnson & Stolzing, 2019) were identified. We selected the following 20 proteins to highlight that prominently impact longevity and/or age‐related disease when manipulated in an animal model: ADAMTS5, BDNF, CCL11, CGA.FSHB, FGA.FGB.FGG, IL15RA, IL6, LIFR, LILRB2, MMP12, NAB1, NTN1, PAK4, PLA2G2A, PLXNB2, POMC, PRKAA1.PRKAB1.PRKAG1, RBM3, SIRT5, and UFM1. Interesting literature connections for these proteins are listed in Table 1 and graphs visualizing how the expression level of these proteins changes with age are shown in Figure S1.

**Table 1:** 20 examples of common aging plasma proteins with highly intriguing links to aging and/or disease

Protein	*q*‐value, age coefficient	Intriguing connections to aging and/or disease
ADAMTS5	7.69E−65, 1.88E−03	Mice lacking *Adamts5* are protected from cartilage destruction following joint instability induced by surgery (Glasson et al., 2005) *ADAMTS5* is overexpressed in osteoarthritic cartilage from mice and humans (Lin et al., 2009) Wwp2 promotes the maintenance of cartilage homeostasis via the suppression of Adamts5 in mice (Mokuda et al., 2019)
BDNF	2.78E−30, 2.84E−03	Treating Huntington's disease mice with human mesenchymal stem cells that overexpress BDNF extends life span and increases neurogenesis‐like activity (Pollock et al., 2016) Exercise elevates BDNF levels and induces adult hippocampal neurogenesis in Alzheimer's disease mice (S. H. Choi et al., 2018) In a zebrafish model of Alzheimer's disease, BDNF enhances neurogenesis and neural stem cell plasticity (Bhattarai et al., 2020)
CCL11	8.87E−94, 3.34E−03	In a cohort of non‐diabetic women, plasma levels of CCL11 are associated with central obesity and are reduced in response to an exercise program (Choi et al., 2007) Injecting recombinant Ccl11 into young mice reduces neurogenesis and impairs both memory and learning (Villeda et al., 2011) Administering recombinant Ccl11 to young mice results in synaptic loss and increased microglial reactivity (Das et al., 2019)
CGA.FSHB	2.89E−320, 1.64E−02	Long‐lived mice deficient in growth hormone receptor exhibit decreased plasma levels of follicle‐stimulating hormone (V. Chandrashekar et al., 2007) Bone loss is mitigated in ovariectomized mice treated with an antibody specific to the β‐subunit of follicle‐stimulating hormone (Zhu et al., 2012) An antibody specific to the β‐subunit of follicle‐stimulating hormone decreases body fat, stimulates brown adipose tissue, and promotes thermogenesis in mice (Liu et al., 2017)
FGA.FGB.FGG	8.38E−11, 7.25E−04	Treating mice with fibrinogen causes demyelination via the induction of adaptive immune responses and the recruitment of peripheral macrophages (Ryu et al., 2015) Inhibiting fibrin with the monoclonal antibody 5B8 attenuates neurodegeneration and innate immunity in mouse models of multiple sclerosis and Alzheimer's disease (Ryu et al., 2018) In Alzheimer's disease mice, genetically deleting a binding motif in fibrinogen reduces neuroinflammation and cognitive decline (Merlini et al., 2019)
IL15RA	1.31E−43, 1.57E−03	Mice lacking *Il15ra* have a higher body temperature, consume more oxygen, and are leaner despite increased food intake (He et al., 2010) Fast skeletal muscles in *Il15ra* ^−/−^ mice are more resistant to fatigue and have a greater exercise capacity (Pistilli et al., 2011) *Il15ra* ^−/−^ mice are protected from diet‐induced obesity and exhibit enhanced fatty acid oxidation (Loro et al., 2015)
IL6	4.13E−05, 7.16E−04	The ability to ward off bacterial or viral infection is impaired in *Il6* knockout mice (Kopf et al., 1994) Genetically disrupting *Il6* in mice impairs liver regeneration and causes liver failure (Cressman et al., 1996) Transgenic mice overexpressing human *IL6* are substantially smaller and have reduced levels of circulating Igf1 (De Benedetti et al., 1997)
LIFR	5.43E−08, −6.27E−04	Increasing the expression of *LIFR* in malignant cells suppresses tumor metastasis in mice (D. Chen et al., 2012) Inoculating mice with breast cancer cells lacking *LIFR* promotes bone destruction (R. W. Johnson et al., 2016) Mouse Lifr contains separate protein domains that either maintain stem cell self‐renewal or induce differentiation (X. J. Wang et al., 2017)
LILRB2	9.22E−21, 1.07E−03	The genetic deletion of *Lilrb3* (mouse ortholog of human *LILRB2*) protects mice from Aβ‐induced memory impairment (Kim et al., 2013) Small molecule inhibitors targeting the binding site of LILRB2 disrupt LILRB2‐Aβ interactions and reduce Aβ cytotoxicity (Cao et al., 2018) The anti‐tumor effects of T‐cell immune checkpoint inhibitors are enhanced by the blockade of LILRB2 (Chen et al., 2018)
MMP12	2.53E−92, 3.64E−03	A single nucleotide polymorphism in *MMP12* is associated with a reduced risk of chronic obstructive pulmonary disease (Hunninghake et al., 2009) Large artery atherosclerosis is associated with a genetic variant in the *MMP12* locus and this gene is overexpressed in carotid plaques (Traylor et al., 2014) In mice deficient in *Ldlr*, the deletion of *Mmp12* protects male mice from both arterial stiffness and atherosclerosis (Liu et al., 2019)
NAB1	1.14E−26, −2.01E−03	NAB1 is upregulated in human heart failure and mice overexpressing *Nab1* are protected from induced hypertrophy (Buitrago et al., 2005) In dogs with moderate heart failure, treatment with rosuvastatin reduces the expression of NAB1 in left ventricular tissue (Zaca et al., 2012) A single nucleotide polymorphism in *NAB1* is associated with systemic lupus erythematosus, rheumatoid arthritis, systemic sclerosis, and idiopathic inflammatory myopathies (Acosta‐Herrera et al., 2019)
NTN1	2.09E−50, 2.32E−03	Overexpressing *Ntn1* in the mouse gut suppresses intestinal cell apoptosis and promotes tumor development (Mazelin et al., 2004) In mice lacking the low‐density lipoprotein receptor, deleting *Ntn1* in macrophages attenuates atherosclerosis (van Gils et al., 2012) In a mouse model of obesity, the hematopoietic deletion of *Ntn1* enhances insulin sensitivity and decreases inflammation (Ramkhelawon et al., 2014)
PAK4	2.47E−04, 9.28E−04	Knocking down *PAK4* in ovarian cancer cells prior to inoculation impedes tumor growth and dissemination in nude mice (Siu et al., 2010) Overexpressing or depleting *Pak4* in mice promotes or delays mammary cancer, respectively (Costa et al., 2019) Growth is suppressed and invasive potential is decreased by the inhibition of *PAK4* in human bladder cancer cells (D. S. Chandrashekar et al., 2020)
PLA2G2A	1.56E−03, 7.11E−04	The size and multiplicity of intestinal tumors are reduced in mice overexpressing *Pla2g2a* (Cormier et al., 1997) The expression of *PLA2G2A* is positively correlated with survival in patients with gastric adenocarcinoma (Leung et al., 2002) In *Muc2* ^−/−^ mice, the transgenic expression of *Pla2g2a* suppresses intestinal tumorigenesis (Fijneman et al., 2008)
PLXNB2	9.33E−40, 1.17E−03	Inhibiting PLXNB2 suppresses the development of xenograft tumors in mice (Yu et al., 2017) Inhibiting PLXNB2 makes prostate cancer stem cells more sensitive to chemotherapy (Li et al., 2020) Motor sensory recovery following spinal cord injury is impaired in mice lacking *Plxnb2* in myeloid cells (X. Zhou et al., 2020)
POMC	1.53E−07, 9.34E−04	Mutations in *POMC* cause early‐onset obesity and adrenal insufficiency in humans (Krude et al., 1998) Blocking the expression of *Pomc* in hypothalamic neurons causes hyperphagia and obesity in mice (Bumaschny et al., 2012) In obese patients with defects in *POMC*, treatment with a melanocortin‐4 receptor agonist reduces hunger and induces weight loss (Kuhnen et al., 2016)
PRKAA1.PRKAB1.PRKAG1	4.11E−02, 3.24E−04	Worms constitutively expressing *aakg*‐*2* (worm ortholog of *PRKAG1*) are more resistant to oxidative stress and live longer (Greer et al., 2007) Ampk elevates cellular NAD^+^ levels and enhances the activity of Sirt1 in mouse skeletal muscle (Canto et al., 2009) Overexpressing *AMPKα* (fly ortholog of *PRKAA1*) in neurons induces autophagy and extends life span in *Drosophila* (Ulgherait et al., 2014)
RBM3	6.61E−20, 2.21E−03	Cold stress increases the expression level of *RBM3* in multiple different human cell lines (Danno et al., 1997) Overexpressing *Rbm3* prevents neuronal loss and prolongs survival in Alzheimer's disease mice (Peretti et al., 2015) In response to hypoxic ischemia, Rbm3 promotes the proliferation of neural stem/progenitor cells in the subgranular zone (X. Zhu et al., 2019)
SIRT5	9.61E−10, 8.53E−04	Creating a *Sirt5* deficiency in Parkinson's disease mice exacerbates motor deficits and dopaminergic degeneration (Liu et al., 2015) Knocking out *Sirt5* in mice leads to the development of hypertrophic cardiomyopathy (Sadhukhan et al., 2016) Mice deficient in *Sirt5* exhibit cold intolerance and a reduced browning capacity in white adipose tissue (Shuai et al., 2019)
UFM1	2.51E−03, 5.82E−04	Deletion mutations that affect the ufm‐1 cascade result in reduced fecundity and life span in worms (Hertel et al., 2013) RNAi knockdown against *Ufm1* decreases life span and causes locomotive defects in fruit flies (Duan et al., 2016) A homozygous mutation in *UFM1* causes early‐onset encephalopathy with progressive microcephaly in humans (Nahorski et al., 2018)

For each protein, the *q*‐value and age coefficient (measured in a human proteomic dataset derived from 4263 individuals aged 18–95 years) as well as three relevant connections to aging and/or disease are provided.

### 2.3 A large proportion of common aging plasma proteins affect animal life span

Among the literature connections identified for all of our common aging plasma proteins (Table S2), at least 64 proteins (12.1% of all 529 proteins) increase or decrease life span when manipulated in normal animal models. 35 of these 64 proteins affect life span in a vertebrate model. The number of life span regulators is expanded to 108 (20.42% of all 529 proteins) when disease models, stress models, and models harboring multiple different genetic alterations are included. The following nine proteins were found to significantly extend life span when manipulated in normal, non‐diseased mice or fish: AKT2, GDF11, GDF15, GHR, NAMPT, PAPPA, PLAU, PTEN, and SHC1. Vertebrate life extension details for all nine of these proteins are provided in Table 2 and graphs visualizing how the expression level of these proteins changes with age are shown in Figure S2.

**Table 2:** Examples of common aging plasma proteins that can significantly extend life span in a vertebrate animal model when manipulated

Protein	*q*‐value, age coefficient	Life span effect
AKT2	1.61E−16, 1.04E−03	Mice deficient in *Akt2* display a **9.1%** increase in **median** survival and an improvement in myocardial contractile function (Ren et al., 2017)
GDF11	1.92E−02, −7.20E−04	In killifish, levels of gdf11 decrease with age and treating aged animals with recombinant gdf11 lengthens **mean** life span by **8.3%** (Zhou et al., 2019)
GDF15	1.71E−249, 5.26E−03	The overexpression of human GDF15 in female mice extends **median** life span (**19.5%** for transgenic line 1377 and **12.9%** for transgenic line 1398) and protects against weight gain and insulin insensitivity (Wang et al., 2014)
GHR	7.56E−24, −1.53E−03	*Ghr* ^−/−^ mice live longer (**8.7%–28.2%** increase in **median** life span depending on the sex and mouse strain), weigh less, and exhibit reduced levels of fasting glucose and insulin (Coschigano et al., 2003)
NAMPT	5.39E−04, 1.12E−03	Wheel‐running activity is enhanced and longevity is boosted (**10.2%** increase in **median** life span) in aged female mice treated with extracellular vesicles containing Nampt (Yoshida et al., 2019)
PAPPA	9.29E−05, 8.09E−04	The incidence of spontaneous tumors is reduced and life is prolonged (**37.5%** increase in **mean** life span) in mice lacking *Pappa* (Conover & Bale, 2007)
PLAU	6.46E−11, 8.67E−04	Overexpressing *Plau* in mice elongates **median** life span (**36%**, **16%**, and **23%** for 75th, 50th, and 25th percentile survivors, respectively), reduces food intake, and decreases body weight (Miskin & Masos, 1997)
PTEN	2.41E−02, 4.06E−04	Longevity is enhanced (**12.4%** increase in **median** life span), cancer incidence is decreased, and insulin sensitivity is improved in mice harboring additional copies of *Pten* (Ortega‐Molina et al., 2012)
SHC1^a^	7.18E−04, 8.53E−04	**Median** life span is extended by **27.9%** and oxidative stress resistance is enhanced in *Shc1* ^−/−^ mice (Migliaccio et al., 1999)

For each protein, the *q*‐value and age coefficient (measured in a human proteomic dataset derived from 4263 individuals aged 18–95 years) as well as the life span effect are included. Bolded words and numbers highlight the lifespan effect in response to a given intervention.

^a^ A follow‐up study assessed life span in *Shc1* knockout mice at two different locations. At one location, *Shc1* ^−/−^ mice on a 40% calorie restriction diet exhibited a survival benefit (**median** 70th percentile survival was increased by **8%**). At the other site, no longevity benefit was observed in *Shc1* knockout mice fed ad libitum (Ramsey et al., 2014).

### 2.4 Well‐known anti‐aging drugs and interventions are implicated by our common aging plasma proteins

Many of our 529 common aging plasma proteins were also implicated by established anti‐aging drugs and interventions (Table S2), including glycine (Miller et al., 2019), rapamycin (Bitto et al., 2016), spermidine (Eisenberg et al., 2016), nicotinamide riboside (Zhang et al., 2016), metformin (Kulkarni et al., 2020), caloric restriction (Most et al., 2017), intermittent fasting (de Cabo & Mattson, 2019), and exercise (Garatachea et al., 2015). These connections prompted us to analyze our identified vertebrate longevity proteins in the GLAD4U drug database (Jourquin et al., 2012). For our nine vertebrate life extension proteins, the three enriched terms were “insulin recombinant,” “somatropin recombinant,” and “egfr inhibitors” (Figure S3A). Among the enriched terms for all 35 vertebrate longevity proteins was the immunosuppressant “sirolimus,” which is another name for rapamycin (Figure S3B). Other aging‐relevant enriched drug terms included “cardiovascular system” as well as the anti‐cancer drugs “doxorubicin” and “erlotinib” (Figure S3B).

### 2.5 Diverse processes pertinent to the immune system are strongly implicated by plasma proteins that trend toward an increased expression level with age

**Figure 2:**
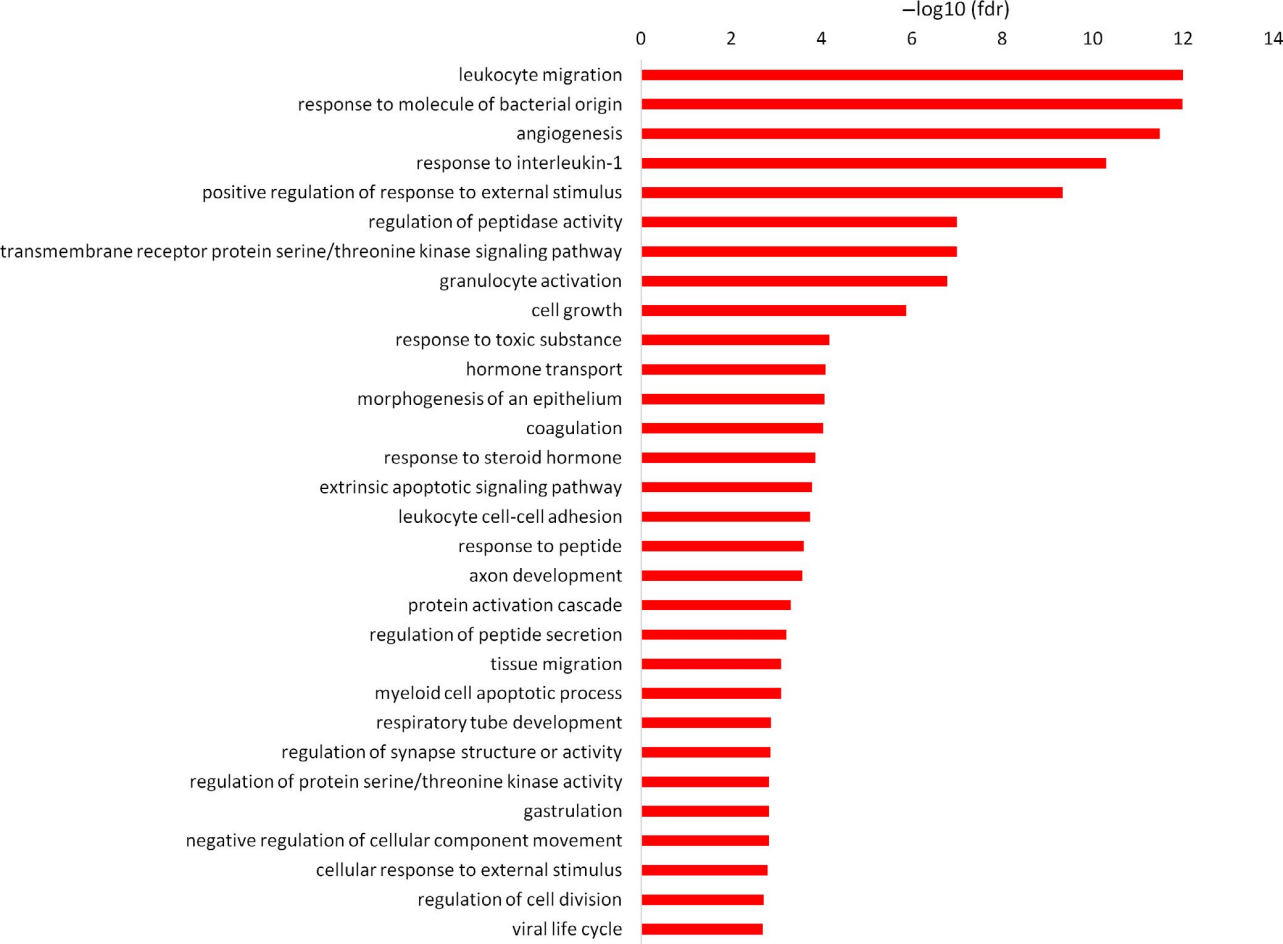
An overrepresentation analysis in the Gene Ontology Biological Process database was performed for all proteins that significantly (*q* < 0.05) change their expression level with age in human plasma and have a positive age coefficient. The top 30 enrichment results are presented as –log10(fdr)

We next performed enrichment analyses in the Gene Ontology Biological Process (GO BP) database (The Gene Ontology, 2019) for different sets of proteins. For the proteins that significantly trend toward increased expression with age, a very prominent theme of the immune system was apparent. Among the top 30 GO BP terms (Figure 2), the following six terms relevant to the immune system were identified: “leukocyte migration,” “response to molecule of bacterial origin,” “response to interleukin‐1,” “granulocyte activation,” “leukocyte cell‐cell adhesion,” and “viral life cycle.” The proteins that significantly trend toward decreased expression with age were associated with the following enriched terms: “positive regulation of response to external stimulus,” “protein activation cascade,” “protein kinase B signaling,” “extracellular structure organization,” and “neutrophil mediated immunity” (Figure S4A).

For the plasma proteins that can impact longevity in normal animals, the enriched terms were quite diverse (Figure S4B). Themes of nutrient intake and metabolism (i.e., “response to nutrient levels,” “regulation of carbohydrate metabolic process,” and “response to ketone”) and the immune system (i.e., “response to transforming growth factor beta” and “neutrophil mediated immunity”) were present. Terms relevant to protein homeostasis (i.e., “positive regulation of proteolysis”) and stress resistance (i.e., “response to oxidative stress”) were also identified (Figure S4B). For the larger list of proteins that can impact longevity in any animal model, we collated the top 30 GO BP terms (Figure S5). Prominent themes pertinent to cell movement, cell growth and proliferation, the immune system, and the circulatory system were identified (Figure S5).

### 2.6 Machine‐learning analyses uncover numerous aging clocks reflecting different aspects of aging

Having established that these common plasma proteins have important connections to aging and disease, we were curious if different protein combinations could be utilized to accurately predict human age. To do this, we tested different clocks in a plasma proteomic dataset derived from 3301 healthy individuals with an age range of 18–76 years. Proteins in this dataset were previously measured using the SOMAscan assay (Sun et al., 2018). We started by testing the following seven clocks: proteins that can extend life span in normal vertebrates, proteins that can modify life span in a normal vertebrate animal model, proteins that can modify life span in a normal animal model, proteins with an entry in the HAGR database, proteins that can modify life span in any animal model (including disease, stress, and genetically complex models), proteins that significantly change their expression level with age, and all common aging plasma proteins (Table S4). We additionally tested the following five clocks based on the top weighted set cover enrichment result (for all 529 proteins) in the Reactome (Jassal et al., 2020), Panther (Mi & Thomas, 2009), KEGG (Kanehisa & Goto, 2000), WikiPathways (Slenter et al., 2018), and GO BP (The Gene Ontology, 2019) databases: proteins associated with “peptide hormone biosynthesis” in Reactome, proteins associated with “plasminogen activating cascade” in Panther, proteins associated with “complement and coagulation cascades” in KEGG, proteins associated with “human complement system” in WikiPathways, and proteins associated with “leukocyte migration” in GO BP (Table S5).

**Figure 3:**
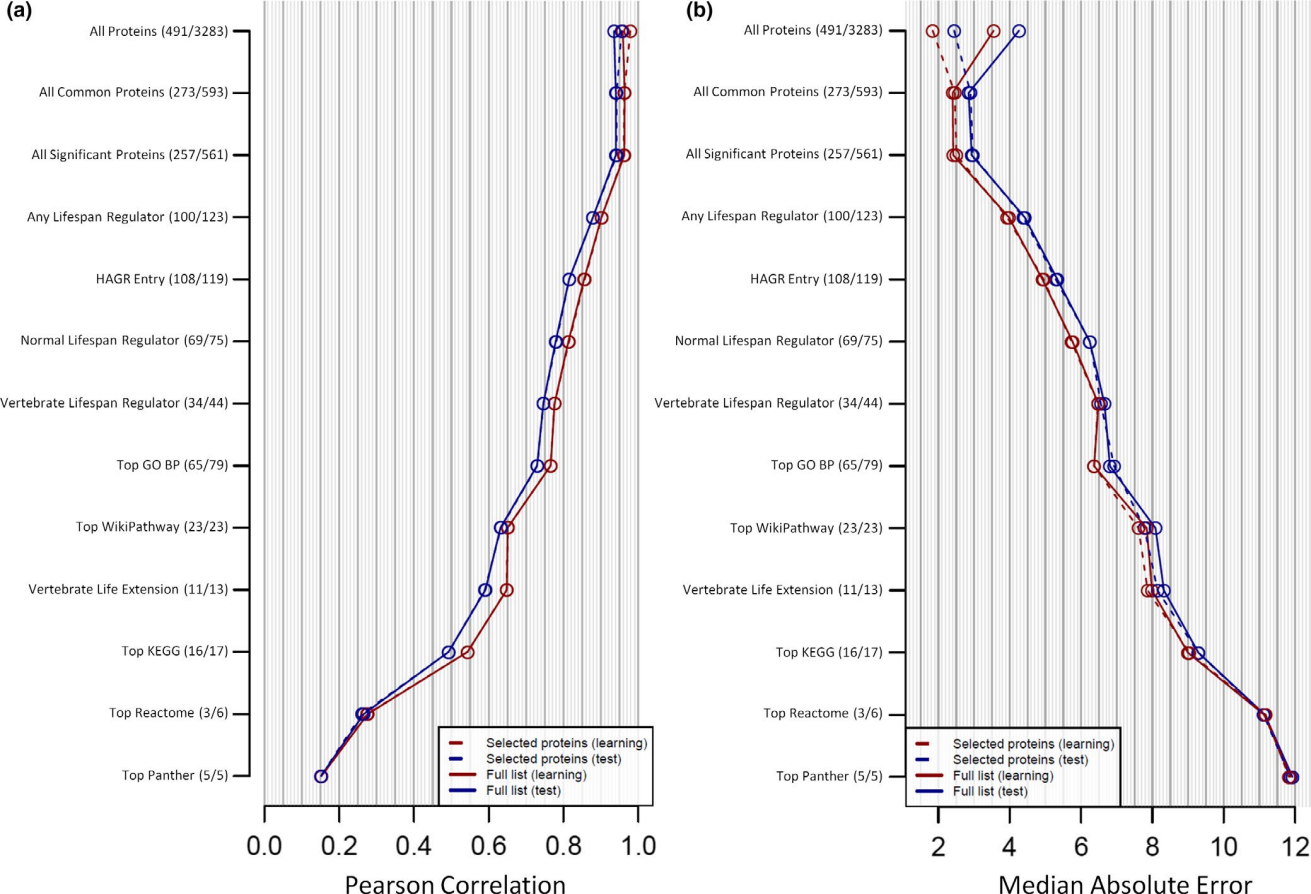
The ability of 13 different protein sets to predict age in a plasma proteomic dataset derived from 3301 human participants (age range of 18–76 years) was tested using machine learning. For each clock, the learning set utilized 2178 subjects and the test set utilized 1123 subjects. LASSO modeling was also performed for each clock to determine if a smaller set of proteins within the larger set could accurately predict human age. For each of these clocks, the Pearson correlation (a) and median absolute error (b) are reported. The two numbers in parenthesis for each clock indicate the number of available SOMAmers used for the subset of proteins identified by LASSO modeling or the full list of proteins

The Pearson correlation for predicted vs. actual age (Figure 3a) and the median absolute error (MAE) (Figure 3b) for all 12 of these clocks is shown. For each clock, two‐thirds of the dataset (*n* = 2178) was used for the training model and one third of the dataset (*n* = 1123) was used for the validation model. We also fitted a LASSO model for each clock to determine if there was a subset of highly predictive proteins within the full protein list. We additionally compared these results to a clock comprised of all 2978 proteins available for measurement in our plasma proteomic dataset. Detailed information for each clock is provided in Table S6.

Of our 12 proposed plasma proteomic aging clocks (Tables S4 and S5), the most predictive clock received all common aging plasma proteins as the input. For this clock, the learning set had a Pearson correlation of 0.96 and the test set had a Pearson correlation of 0.94 (Figure 3a). The respective MAE values for the learning and tests sets were 2.4 and 2.85 years (Figure 3b). The clock comprised of all significant proteins was a close second with a Pearson correlation of 0.96 in the learning set (Figure 3a), a Pearson correlation of 0.94 in the test set (Figure 3a), a MAE of 2.42 years in the learning set (Figure 3b), and a MAE of 2.93 years in the test set (Figure 3b). Clocks comprised of proteins that regulate life span in any animal model or have a HAGR entry had a Pearson correlation >0.8 in the test set (Figure 3a). Proteins that either impact longevity in any normal animal model, affect life span in a normal vertebrate model, or make up the top GO BP pathway result had a Pearson correlation >0.7 in the test set while the proteins that make up the top WikiPathways result had a Pearson correlation >0.6 in the test set (Figure 3a). The proteins capable of extending life span in a normal vertebrate animal model had a Pearson correlation of 0.65 in the learning set and 0.59 in the test set (Figure 3a). The least predictive clocks were the top KEGG, Reactome, and Panther results, which had a respective Pearson correlation of 0.49, 0.27, and 0.15 in the test set. For all measurements, the Pearson correlation ranged from 0.15 to 0.98 (Figure 3a) and the MAE ranged from 1.84 to 11.93 years (Figure 3b). Clock accuracy positively correlated with the number of SOMAmer inputs (Figure S6). Two examples of more minimalistic aging clocks—proteins that regulate life span in any animal model or proteins that regulate life span in a normal vertebrate animal model—are shown in Figure S7.

**Figure 4:**
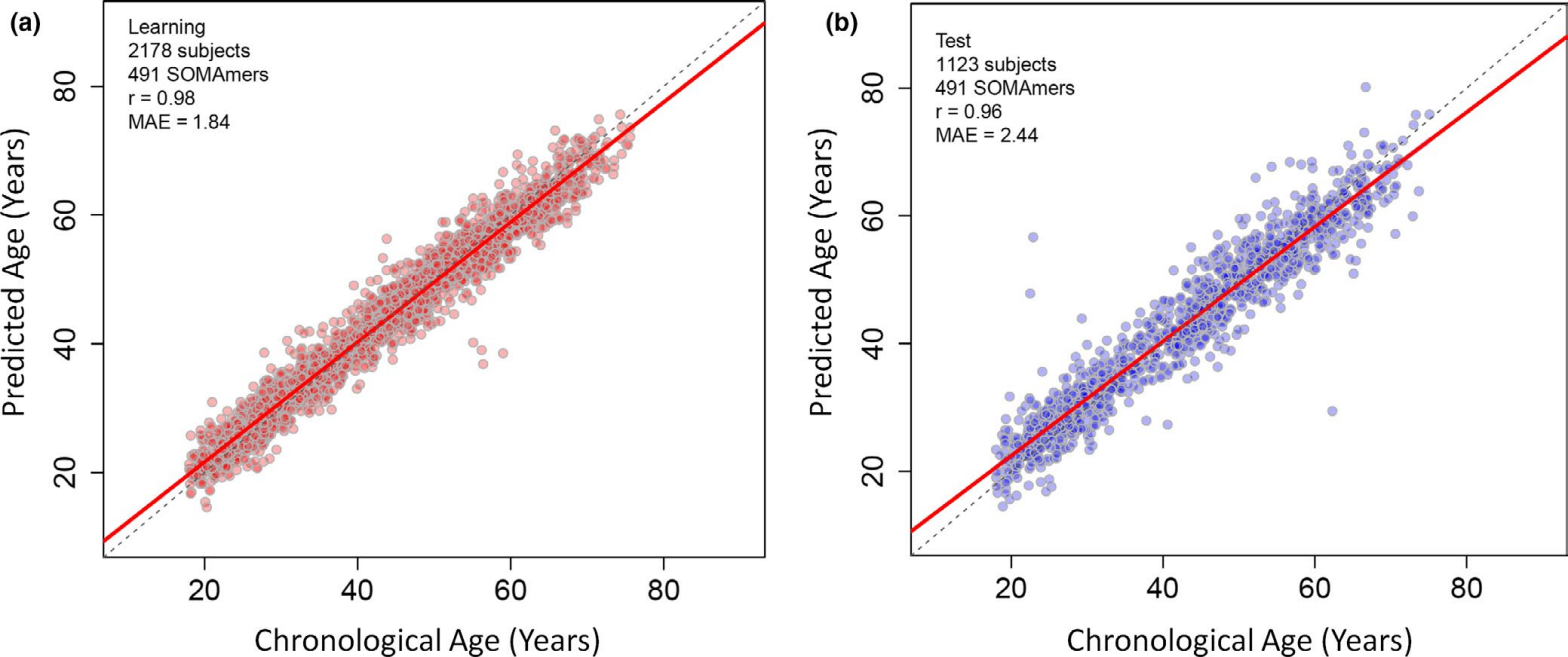
Plots of predicted age vs. chronological age are shown for the most predictive aging clock identified. The most accurate aging clock was identified by LASSO modeling of all 2978 proteins available for measurement in the plasma proteomic dataset derived from 3301 human participants (age range of 18–76 years). This clock used 491 SOMAmers, had a Pearson correlation of 0.98 in the learning set (a), a median absolute error of 1.84 years in the learning set (a), a Pearson correlation of 0.96 in the test set (b), and a median absolute error of 2.44 years in the test set (b). 2178 subjects were utilized for the learning set (a) and 1123 subjects were utilized for the test set (b). MAE = median absolute error

The most predictive clock was identified by LASSO model testing of all 2978 proteins available for measurement. This clock, which utilized 491 SOMAmers, had a Pearson correlation of 0.98 and a MAE of 1.84 years in the learning set (Figure 4a) as well as a Pearson correlation of 0.96 and a MAE of 2.44 years in the test set (Figure 4b). We additionally provide the SOMAmer name, UniProt ID, gene name, and protein name for each component of our most predictive clock in Table S7. Intercept and coefficient information is provided in Table S8. The set of 491 protein entries that make up this ultra‐accurate clock contains multiple common aging plasma proteins that are direct regulators of aging and health (Table S2), such as ADAMTS5, CCL11, GDF15, LEP, and SOD3. Out of the 491 protein entries that make up this clock, a total of 102 (20.77%) contained a common aging plasma protein. For those entries that did not contain a common aging plasma protein, several were direct regulators of animal life span—such as the DNA repair protein ERCC1 (de Waard et al., 2010), the glycine‐relevant protein GNMT (Tain et al., 2020), the lipase enzyme LIPN (Johnson, 2020), and the insulin receptor protein (Blüher, 2003). An enrichment analysis of the proteins in this clock heavily implicated various immune and inflammatory processes (Figure S8). This clock is predictive in both men and women (Table S9).

We additionally tested the ability of this ultra‐predictive clock to measure age in two independent plasma proteomic datasets that were previously generated. The first dataset is comprised of 171 individuals with an age range of 21–107 years (Lehallier et al., 2019), and the second dataset is comprised of 47 healthy individuals with an age range of 19–77 years (Santos‐Parker et al., 2018). For the former dataset, the Pearson correlation was 0.9 (Figure S9A). For the latter dataset, the Pearson correlation was 0.91 (Figure S9B). Thus, this clock is able to accurately predict age with a Pearson correlation ≥0.9 in three different human cohorts (Figure 4 and Figure S9).

### 2.7 Physically inactive subjects exhibit a higher predicted age than their chronological age

**Figure 5:**
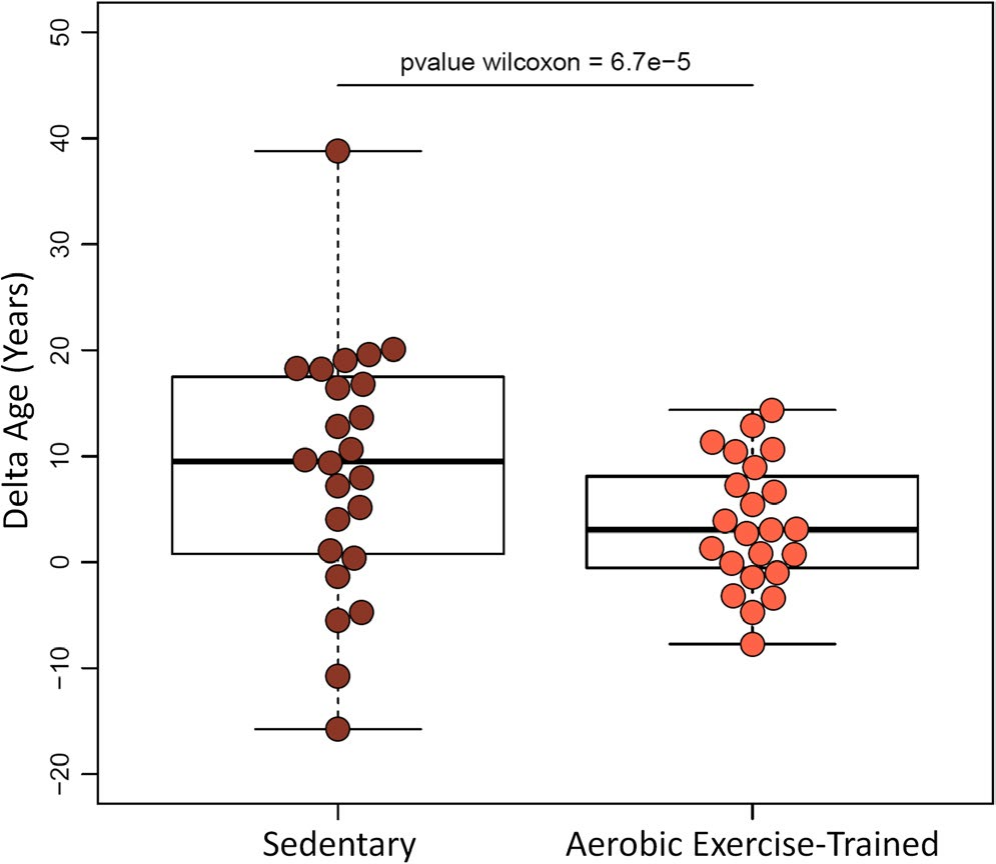
We used our ultra‐predictive aging clock to predict age in a human plasma proteomic dataset containing sedentary subjects as well as individuals that are aerobic exercise‐trained. For sedentary subjects, their respective chronological and predicted ages were 37.54 ± 20.88 and 46.34 ± 26.48 years. For aerobic exercise‐trained subjects, their respective chronological and predicted ages were 37.35 ± 19.82 and 40.91 ± 18.48 years. Results are presented as mean ± standard deviation. The difference in delta age (i.e., the difference between chronological and predicted age) between sedentary and aerobic exercise‐trained subjects was statistically significant (*p*‐value = 6.7E−5)

Previously, Santos‐Parker et al used the SOMAscan assay to measure the plasma proteome in 47 healthy adults (Santos‐Parker et al., 2018). This patient cohort contained individuals that were sedentary as well as individuals that were aerobic exercise‐trained. Using our most predictive clock (Figure 4), we demonstrate that the sedentary individuals from this cohort exhibit a higher predicted age than their chronological age (Figure 5). In contrast, those that are aerobic exercise‐trained displayed a predicted age that was more similar to their chronological age (Figure 5). For sedentary individuals, the respective chronological and predicted ages were 37.54 ± 20.88 and 46.34 ± 26.48 years. For aerobic exercise‐trained individuals, the respective chronological and predicted ages were 37.35 ± 19.82 and 40.91 ± 18.48 years. The delta between chronological and predicted age was significantly different between the sedentary and aerobic exercise‐trained groups (*p*‐value = 6.7E–5). The predicted age difference between aerobic exercise‐trained and sedentary individuals was 5.43 years.

Interestingly, many of the proteins contained in our 491‐entry clock were previously used by Williams et al to generate plasma protein models that can accurately predict various health outcomes (Williams et al., 2019). We found that many of the proteins used to predict the following health outcomes were also present in our highly predictive clock: alcohol consumption, cardiopulmonary fitness, cardiovascular primary event risk, current cigarette smoking, diabetes diagnosis within 10 years, energy expenditure from physical activity, kidney filtration, lean body mass, liver steatosis, percent body fat, and visceral adipose tissue. The specific overlapped proteins for each health outcome predictor are listed in Table S10.

### 2.8 Proteins associated with signal transduction or immune system pathways are especially adept at predicting human age

**Figure 6:**
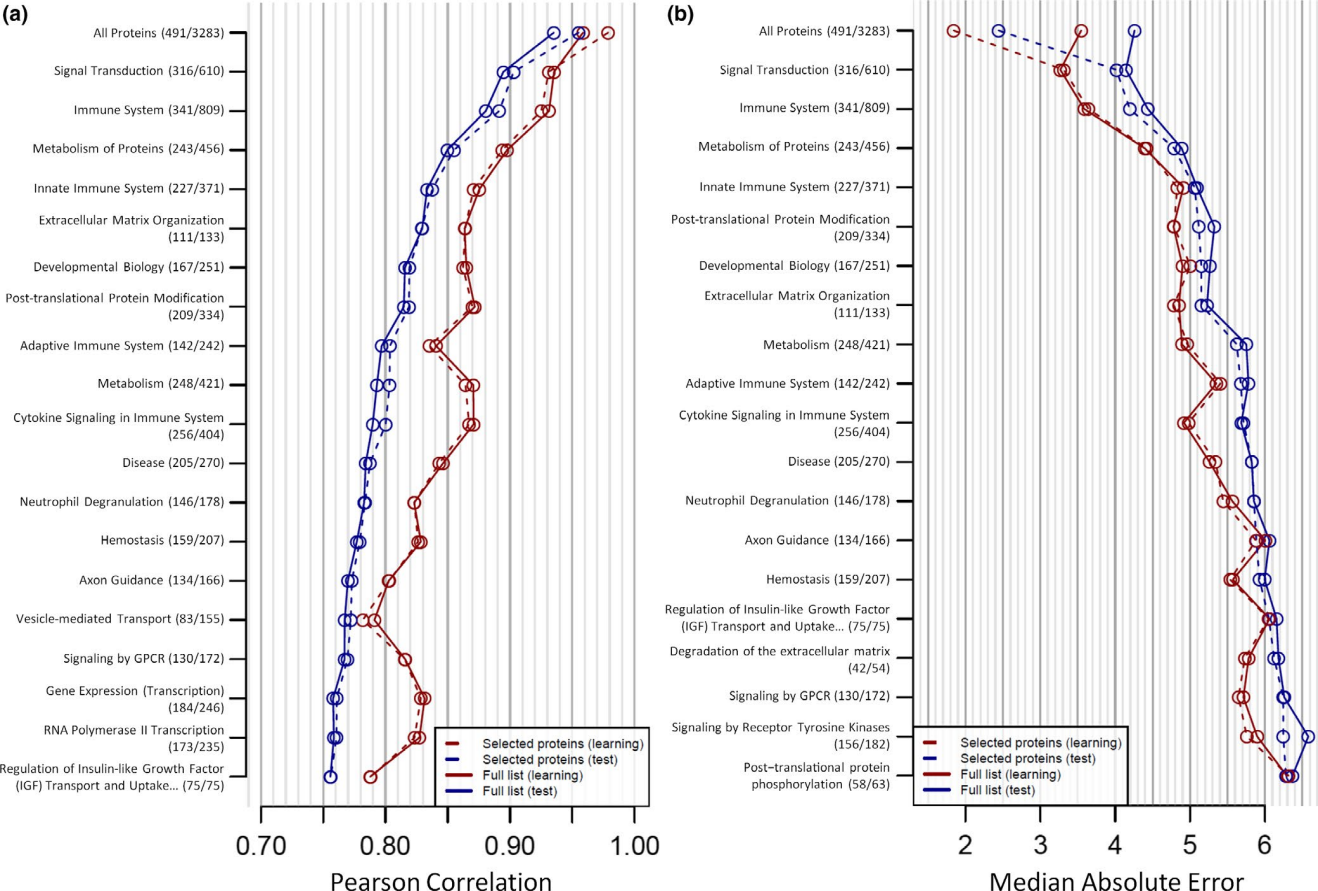
The ability of 1565 protein sets associated with different Reactome pathways to predict age in a plasma proteomic dataset derived from 3301 human participants (age range of 18–76 years) was tested using machine learning. For each clock, the learning set utilized 2178 subjects and the test set utilized 1123 subjects. LASSO modeling was also performed for each clock to determine if a smaller set of proteins within the larger set could more accurately predict human age. We visualize the Pearson correlation (a) for the 19 pathways with the highest Pearson correlation. We also visualize the median absolute error (b) for the 19 pathways with the lowest median absolute error. The two numbers in parenthesis for each clock indicate the number of available SOMAmers used for the subset of proteins identified by LASSO modeling or the full list of proteins. The full name of the pathway abbreviated with ellipses is “Regulation of insulin‐like growth factor (IGF) transport and uptake by insulin‐like growth factor binding proteins (IGFBPs)”

Our aging clock data (Figure 3) demonstrate that some pathways are more capable of predicting human age than others. To test this more comprehensively, we assessed the predictive performance of aging clocks comprised of proteins associated with 1565 different pathways in the Reactome database. Detailed information for each Reactome clock is provided in Table S11. For especially predictive Reactome pathways, we visually show the Pearson correlation (Figure 6a) and/or MAE (Figure 6b). Specifically, we show the 19 pathways with the highest Pearson correlations (Figure 6a) and the 19 pathways with the lowest MAEs (Figure 6b) in the LASSO test sets. The Reactome pathways with the five highest Pearson correlations were as follows: “signal transduction,” “immune system,” “metabolism of proteins,” “innate immune system,” and “extracellular matrix organization.” Among the 19 Reactome pathways with the highest Pearson correlations (Figure 6a), the following five were all immune‐related: “immune system,” “innate immune system,” “adaptive immune system,” “cytokine signaling in immune system,” and “neutrophil degranulation.” The most predictive clock (“signal transduction”) had a Pearson correlation of 0.94 in the learning set and 0.89 in the test set (Figure 6a) as well as a MAE of 3.27 years in the learning set and 4.14 years in the test set (Figure 6b). The “immune system” clock was a close second with a Pearson correlation of 0.93 in the learning set and 0.88 in the test set (Figure 6a) as well as a MAE of 3.59 years in the learning set and 4.44 years in the test set (Figure 6b). Plots of predicted age vs. chronological age for these two clocks are shown in Figure S10.

Out of all 1565 Reactome clocks tested (Table S11), seven had a Pearson correlation >0.8 in the test set, 25 had a Pearson correlation >0.7 in the test set, and 20 had a Pearson correlation >0.6 in the test set. Thus, only a small percentage of Reactome pathways are able to accurately predict human age. Compared to the two most predictive Reactome clocks (Figure S10)—each of which contained over 600 SOMAmers—some of these more accurate clocks were relatively minimalistic. The Reactome pathway “Extracellular matrix organization” utilized 133 SOMAmers and had a Pearson correlation of 0.83 and a MAE of 5.23 years in the test set. A total of nine clocks used less than 100 SOMAmers and had a Pearson correlation >0.7 in the test set. For example, the “Degradation of the extracellular matrix” clock contained 54 SOMAmers and, in the test set, had a Pearson correlation of 0.76 and a MAE of 6.18 years. While less accurate, another interesting outlier was the “Negative regulation of TCF‐dependent signaling by WNT ligand antagonists” clock, which contained 8 SOMAmers and had a Pearson correlation of 0.63 and a MAE of 8.07 years in the test set.

## 3 DISCUSSION

In the present study, we discover a novel, ultra‐predictive clock comprised of 491 SOMAmers. Compared to a much larger array of existing aging clocks recently collated by Galkin et al. (2020a), this protein clock is especially predictive. This clock was capable of accurately predicting human age in three different plasma proteomic datasets and was used to demonstrate that physically inactive patients have a much higher predicted age than their chronological age. In contrast, patients that engage in frequent aerobic exercise exhibited a predicted age that was more similar to their chronological age. Since exercise is one of the most effective anti‐aging interventions (Garatachea et al., 2015), these data suggest that this plasma protein age predictor can capture aspects of patient health. Moreover, we unveiled a multitude of novel aging clocks that are made up of a smaller set of proteins. Since proteomics screening can be quite costly (Graham et al., 2005), the ability to predict human age using a minimal set of proteins obviates a financial barrier to performing aging clock measurements. It also makes the prediction of patient age logistically much simpler and therefore more conducive to widespread use. We additionally demonstrate that proteins tangibly associated with different aspects of aging (e.g., proteins that impact animal longevity, proteins that change their expression level with age, or proteins with a listing in the HAGR database) are able to robustly predict human age.

In total, we tested 13 custom clocks and 1565 different Reactome pathway clocks. While our data make it clear that the accuracy of a given clock is correlated with the number of protein entries used, there were several notable exceptions. For example, a clock comprised of proteins that significantly change their expression level with age (which used 561 SOMAmers) had a higher Pearson correlation and a lower MAE than a clock comprised of all measured proteins (which used 3283 SOMAmers). Thus, while the availability of more proteins tends to increase the predictive power of a given clock, the proteins chosen also influence the overall accuracy.

We additionally found nine proteins that both significantly change their expression level with age in human plasma and extend life span in normal vertebrates when manipulated. More broadly, we were able to identify a tangible connection to aging, disease, and health for all 523 protein entries that were comprehensively analyzed. It is important to note that, while some of these connections demonstrated a direct role in regulating the aging process (e.g., a genetic manipulation which impacts longevity and health span), others were more tangential and loosely associated with aging (e.g., protein expression levels were altered in patients with a specific age‐related disease). Of the connections we highlighted, 19.47% had an entry in the HAGR database and 12.1% were capable of impacting longevity in a normal model organism. The percentage of life span regulators increases to 20.42% when disease, stress, and genetically complex models are included. These findings suggest that, in human plasma, proteins which significantly change their expression level with age are also often proteins that directly impact longevity and age‐related disease. Thus, proteomic aging expression screens in plasma may double as screens for anti‐aging drug targets. Future studies are warranted to determine if any of these aging plasma proteins are viable, safe targets for human health span extension.

Our enrichment analysis revealed that a diverse set of processes relevant to inflammation and the immune system were strongly implicated by proteins that increase their expression level with age in human plasma. Furthermore, we found that proteins associated with immune system enrichment terms are especially adept at predicting human age. These findings corroborate an ever‐growing body of data that intimately link aging with immune system dysfunction (Nikolich‐Zugich, 2018). Atypically long‐lived animals exhibit unique gene change relevant to inflammation (Johnson et al., 2019) and genomic (Shen et al., 2020), transcriptomic (Peters et al., 2015), and proteomic (Tanaka et al., 2018) analyses in humans have all connected immunological changes with aging. Interestingly, our “innate immune system” Reactome clock was almost as predictive as our “immune system” clock, despite containing 438 fewer SOMAmers. This would suggest that the innate immune system is especially pertinent to human aging. With these data in mind, it is quite intriguing that one of the most effective anti‐aging drugs capable of extending life span and health span in mice is rapamycin (Bitto et al., 2016), which is clinically used as an immunosuppressant. Thus, clinical therapies that correct immune dysfunction may be particularly capable of improving human health span.

In summary, we propose and validate a plethora of novel aging clocks that are capable of predicting individual age in a large human cohort. Using the most predictive clock we identified, we show that sedentary subjects have a higher predicted age than their chronological age. We additionally discover that proteins which significantly change their expression level with age in human plasma are frequently direct regulators of age‐related disease and/or life span in animal models. Thus, many of these proteins are worthy of further exploration as potential therapeutic targets for the extension of human health span. We also show that diverse processes relevant to inflammation and the immune system are strongly implicated by aging‐relevant proteins. Future studies should build upon these data to help develop effective anti‐aging therapies that can be safely utilized in the clinic.

## 4 EXPERIMENTAL PROCEDURES

### 4.1 Statistical measurements for common aging plasma proteins

We previously identified 529 proteins that were reported to significantly change their expression level with age by two or more different studies (Johnson et al., 2020). These common aging plasma proteins were analyzed in a plasma proteomic dataset derived from 4263 healthy individuals with an age range of 18–95 years (Lehallier et al., 2019). This 4263‐person dataset reflects the combination of two different cohorts: 3301 individuals from the INTERVAL cohort and 962 individuals from the LonGenity cohort. All plasma proteomes were acquired using the SOMAscan assay. For each protein, the q‐value and age coefficient were measured using an online software tool developed by Lehallier et al (Lehallier et al., 2019). Using this tool, a “Linear” regression line and an “All” subset were chosen to make graphs showing how the expression level of select proteins changes with age in human plasma. When multiple different SOMAmer measurements were available for a given protein entry, the first measurement listed was selected.

### 4.2 Database and literature search for connections relevant to aging and health

For each of our common plasma aging proteins, we performed a comprehensive database and literature search to identify connections relevant to aging and health. This included searching for individual protein entries in the HAGR database (Tacutu et al., 2018). UniProt (UniProt, 2019) was utilized to identify default and alternative name recommendations and Alliance of Genome Resources (Alliance of Genome Resources, 2020) was used to find gene orthologs in different organisms. PubMed was employed to search for protein names in conjunction with the terms “lifespan” and “life span.” Other search combinations included the protein name by itself or in combination with “aging,” “disease,” and/or “survival.”

### 4.3 Overrepresentation analyses

Overrepresentation analyses were performed similarly to before (Johnson et al., 2020) using WebGestalt (Liao et al., 2019). UniProt IDs were provided as the inputs, the background was set to all protein‐coding genes, and the FDR significance level was set to 0.05.

### 4.4 Proteomic aging clock generation

The creation of proteomic aging clocks was performed similarly to before (Johnson et al., 2020; Lehallier et al., 2019). Proteomics measurements (performed using the SOMAscan assay) from 3301 human plasma samples collected during the INTERVAL clinical trial were used to test whether aging proteins can predict chronological age. Participants in the INTERVAL randomized controlled trial (ISRCTN24760606) were recruited with the active collaboration of the National Health Service (NHS) Blood and Transplant (http://www.nhsbt.nhs.uk), which supported fieldwork and other elements of the trial. DNA extraction and genotyping were co‐funded by the National Institute for Health Research (NIHR), the NIHR BioResource (http://bioresource.nihr.ac.uk/), and the NIHR Cambridge Biomedical Research Centre at the Cambridge University Hospitals NHS Foundation Trust. The INTERVAL study was funded by NHS Blood and Transplant (11‐01‐GEN). The academic coordinating center for INTERVAL was supported by core funding from the NIHR Blood and Transplant Research Unit in Donor Health and Genomics (NIHR BTRU‐2014‐10024), the UK Medical Research Council (MR/L003120/1), the British Heart Foundation (RG/13/13/30194), and the NIHR Cambridge Biomedical Research Centre at the Cambridge University Hospitals NHS Foundation Trust. Proteomic assays were funded by the academic coordinating center for INTERVAL and Merck Research Laboratories (Merck & Co.). A complete list of the investigators and contributors to the INTERVAL trial was previously reported (Di Angelantonio et al., 2017). The academic coordinating center would like to thank blood donor center staff and blood donors for participating in the INTERVAL trial. Age ranged from 18 to 76 years with a median age of 45 years (first quartile =31; third quartile =55). 1616 participants were female and 1685 were male. Sample selection, processing, and preparation were detailed previously (Sun et al., 2018).

To analyze the accuracy of the plasma proteome to predict chronological aging and the relative predictive power of specific signatures, we used glmnet (Friedman et al., 2010) and fitted ridge regression models for the different lists of proteins (alpha = 0; 100 lambda tested; “lamda.min” as the shrinkage variable estimated after tenfold cross‐validation). Input variables consisted of *z*‐scaled log10–transformed RFUs (relative fluorescence units) and two‐thirds (*n* = 2178) of the samples were used for training the model. The remaining 1123 samples were used as a validation. In addition, we fitted a LASSO model (alpha = 1) to identify a subset of proteins potentially outperforming the full list.

Altogether, we compared 12 different lists of proteins and 1565 different Reactome pathways targeted by at least 2 SOMAmers (out of 2271 Human Reactome pathways) to the full panel of proteins available for measurement (2978 proteins measured by 3283 SOMAmers). The lists of Human Reactome pathways and corresponding genes were obtained from the reactome.db package (Ligtenberg, 2019) and mapped to UniProt ID using the org.Hs.eg.db package (Carlson, 2019). Prediction accuracy of each model was estimated for the training and validation datasets, separately, using a Pearson correlation coefficient between chronological age and predicted age in addition to the corresponding MAE.

### 4.5 Validation of the ultra‐sensitive proteomic clock in independent cohorts and functional relevance

To validate the ultra‐sensitive plasma proteomic clock in independent cohorts, we used an aging proteomic dataset covering a large life span range (Lehallier et al., 2019) and a dataset investigating the effect of exercise in young and old individuals (Santos‐Parker et al., 2018). In the data generated by Lehallier et al. (Lehallier et al., 2019), the age ranged from 21 to 107 years with a median age of 70 years (first quartile = 58, third quartile = 89; 84 males and 87 females). The samples originated from four different cohorts from the United States and Europe (VASeattle, PRIN06, PRIN09, and GEHA, *N* = 171). RFUs for the 1305 proteins measured in these datasets were log10‐transformed and z‐scored.

In the data generated by Santos‐Parker et al. (2018), 31 young (aged 19–32 years, inactive *n* = 16, aerobic exercise‐trained *n* = 15) and 16 healthy older (aged 55–77 years, inactive *n* = 8, aerobic exercise‐trained *n* = 8) were measured. Of the 47 healthy subjects, 15 were female and 32 were male. The version of the SOMAscan platform used in this study measured 1129 proteins and RFUs were similarly log10‐transformed and z‐scored.

Only a subset of the 491 proteins constituting the ultra‐sensitive proteomic clock was measured in these cohorts: *n* = 150 for the study by Lehallier et al. (2019) and *n* = 115 for the study by Santos‐Parker et al. (2018). No re‐fitting of the model was performed but we applied a correction coefficient that was estimated as follows: First, we predicted chronological age in the learning dataset of the INTERVAL cohort using the coefficients of the 491‐SOMAmer proteomic clock but with only available proteins measured in the independent cohorts. Then, we fitted a linear model between predicted age and chronological age and estimated the correction coefficient to correct for slope offset of each subclock, separately. This correction coefficient was 2.62 for the study by Lehallier et al. (Lehallier et al., 2019) and 4.57 for the study by Santos‐Parker et al. (2018).

To estimate whether aerobic exercise has an effect on aging, we calculated delta age, which corresponds to the difference between predicted age and chronological age, and tested statistical significance using the Wilcoxon signed‐rank test. Finally, we compared the proteins constituting the ultra‐predictive clock with protein predictors of 12 health traits such as smoking, percent body fat, and cardiopulmonary fitness according to a recent study from Williams et al. (2019). To do this, we mapped protein names to gene symbols and estimated the percentage of genes measured in our study that were involved in the aging clock and in the different, previously reported health outcome predictors.

## CONFLICT OF INTEREST

The authors have no conflicts of interest to declare.

## AUTHOR CONTRIBUTIONS

BL performed the proteomic aging clock analyses and measurements, contributed to study design, and contributed to manuscript writing. MNS performed enrichment analyses and edited the manuscript. TW‐C provided mentoring and essential resources for BL as well as reviewed the manuscript. AAJ conceived and designed the study, performed the database and literature review for all common aging plasma proteins, wrote the manuscript, and performed enrichment analyses.

## Supporting information

Fig S7

## Data Availability

Age measurements from the plasma proteomic dataset derived from 4263 individuals (aged 18–95 years) are accessible via an online software tool (https://twc‐stanford.shinyapps.io/aging_plasma_proteome/). The full plasma proteomic dataset derived from 3301 individuals (aged 18–76 years) is available in the European Genotype Archive (accession number EGAS00001002555).
