# Supplementary figures and images for: Data mining of human plasma proteins generates a multitude of highly predictive aging clocks that reflect different aspects of aging
Source: Aging Cell. 2020 Oct 8;19(11):e13256. doi: 10.1111/acel.13256 (PMC7681068; doi:10.1111/acel.13256)

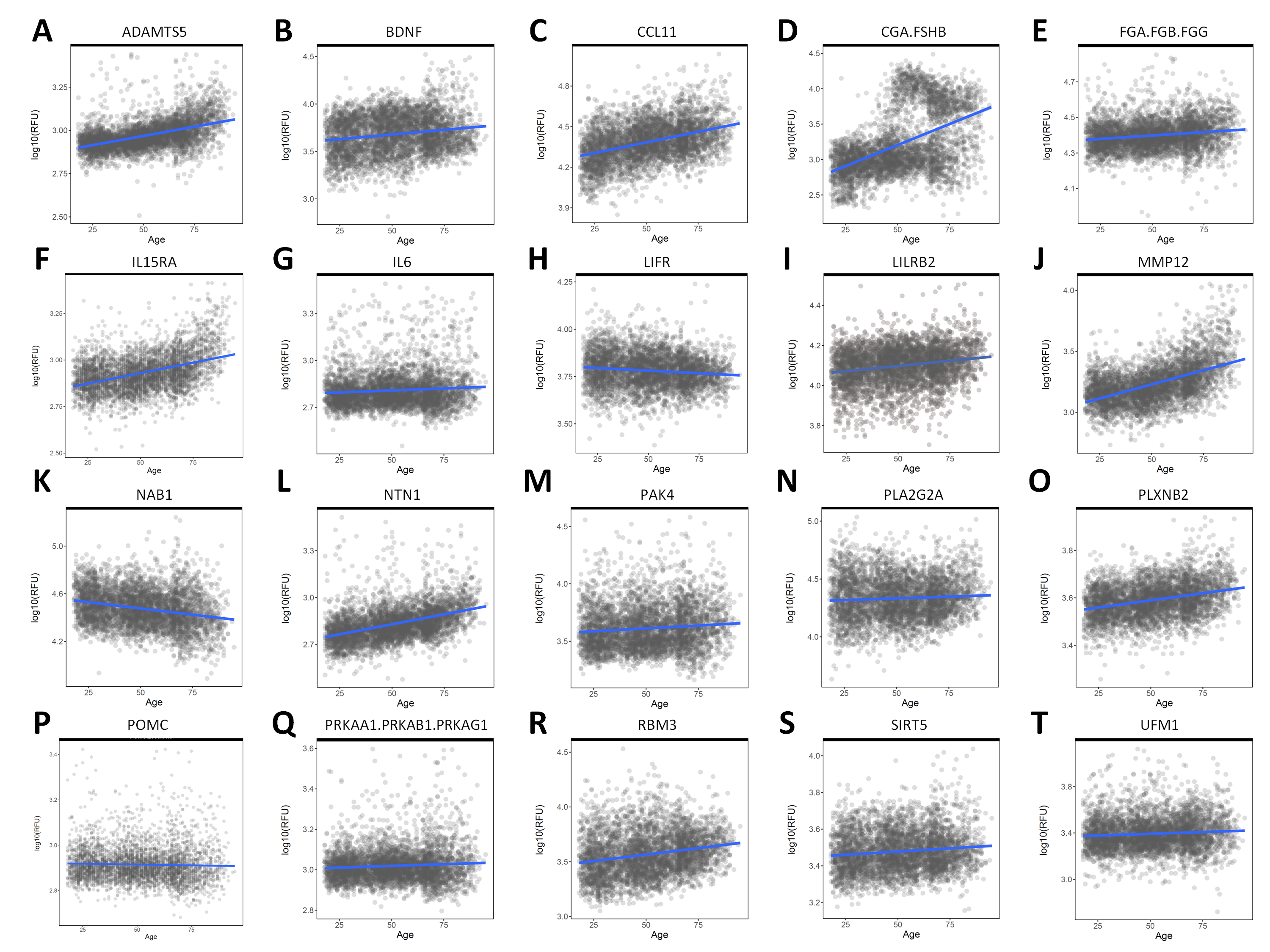

Supplement: Supplementary file 1 [file ACEL-19-e13256-s001.TIF]

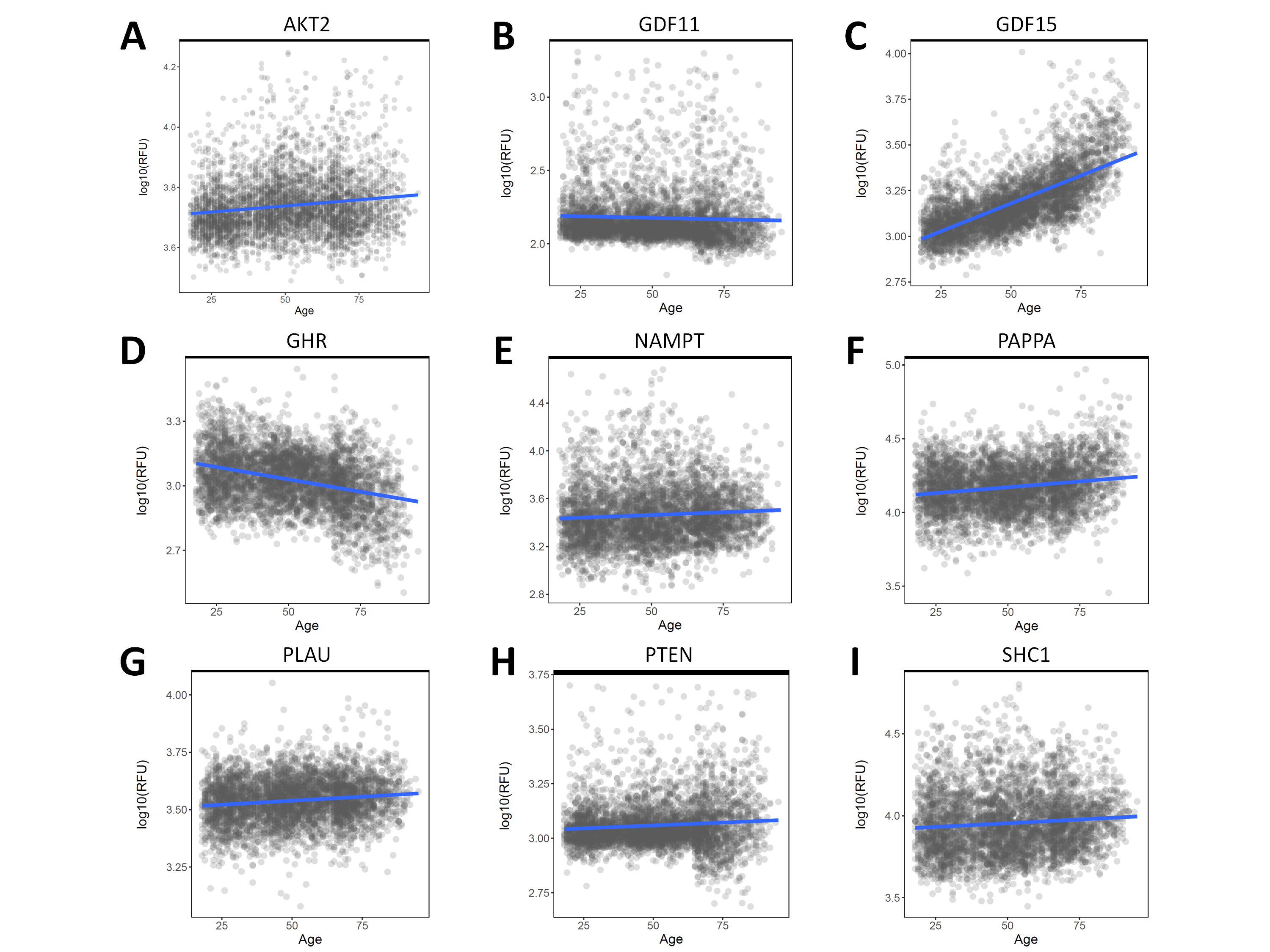

Supplement: Supplementary file 2 [file ACEL-19-e13256-s002.TIF]

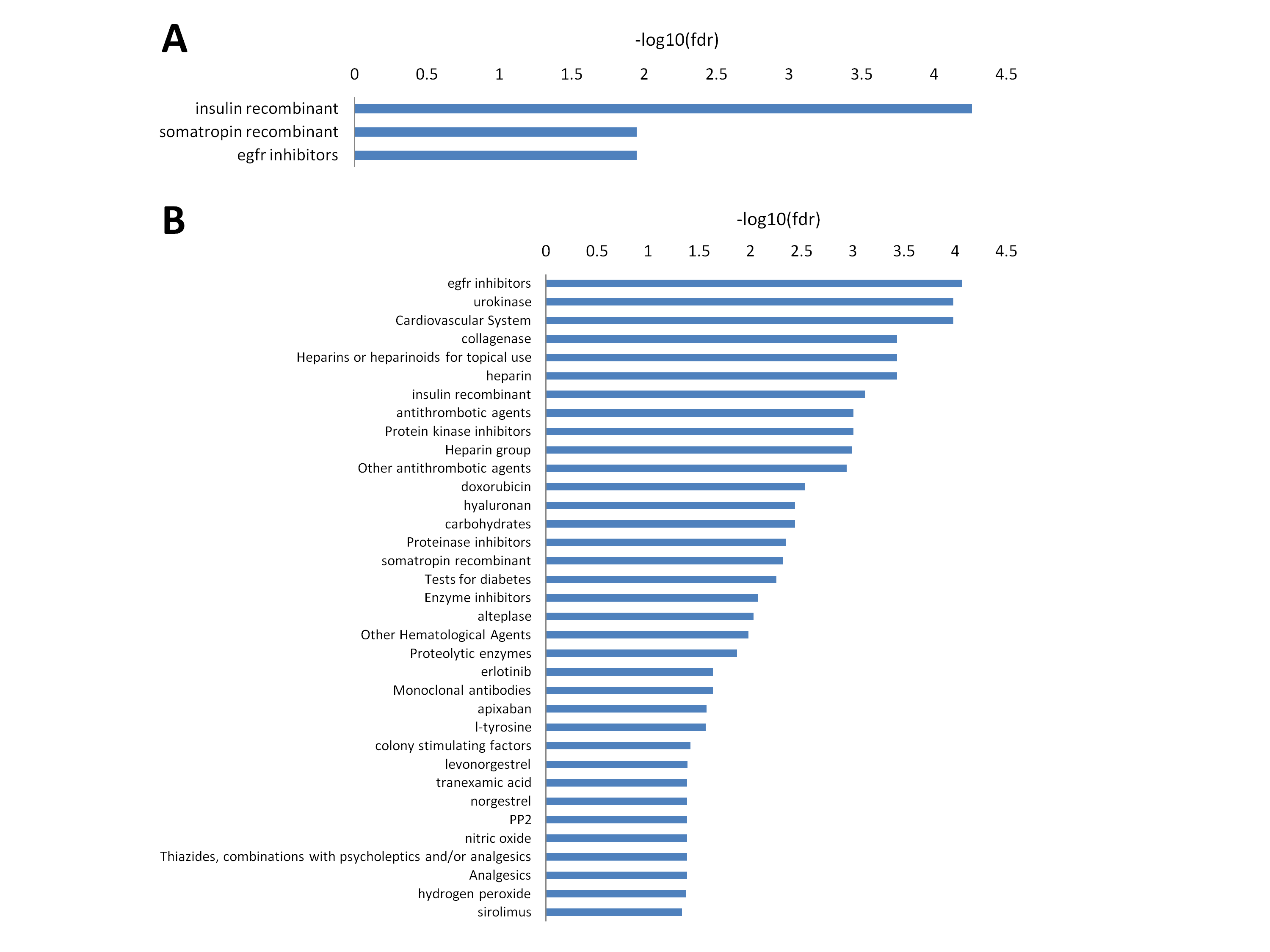

Supplement: Supplementary file 3 [file ACEL-19-e13256-s003.TIF]

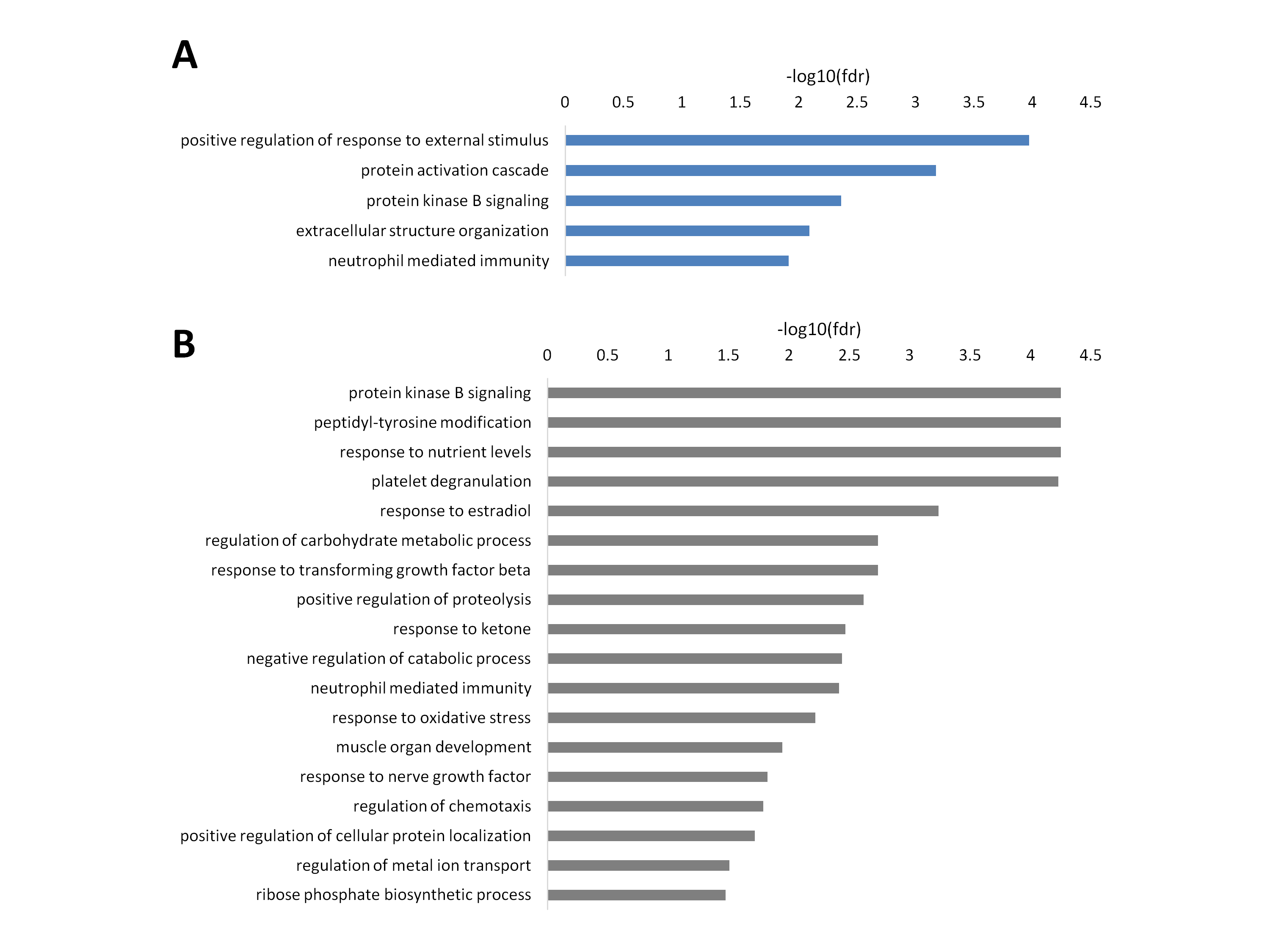

Supplement: Supplementary file 4 [file ACEL-19-e13256-s004.TIF]

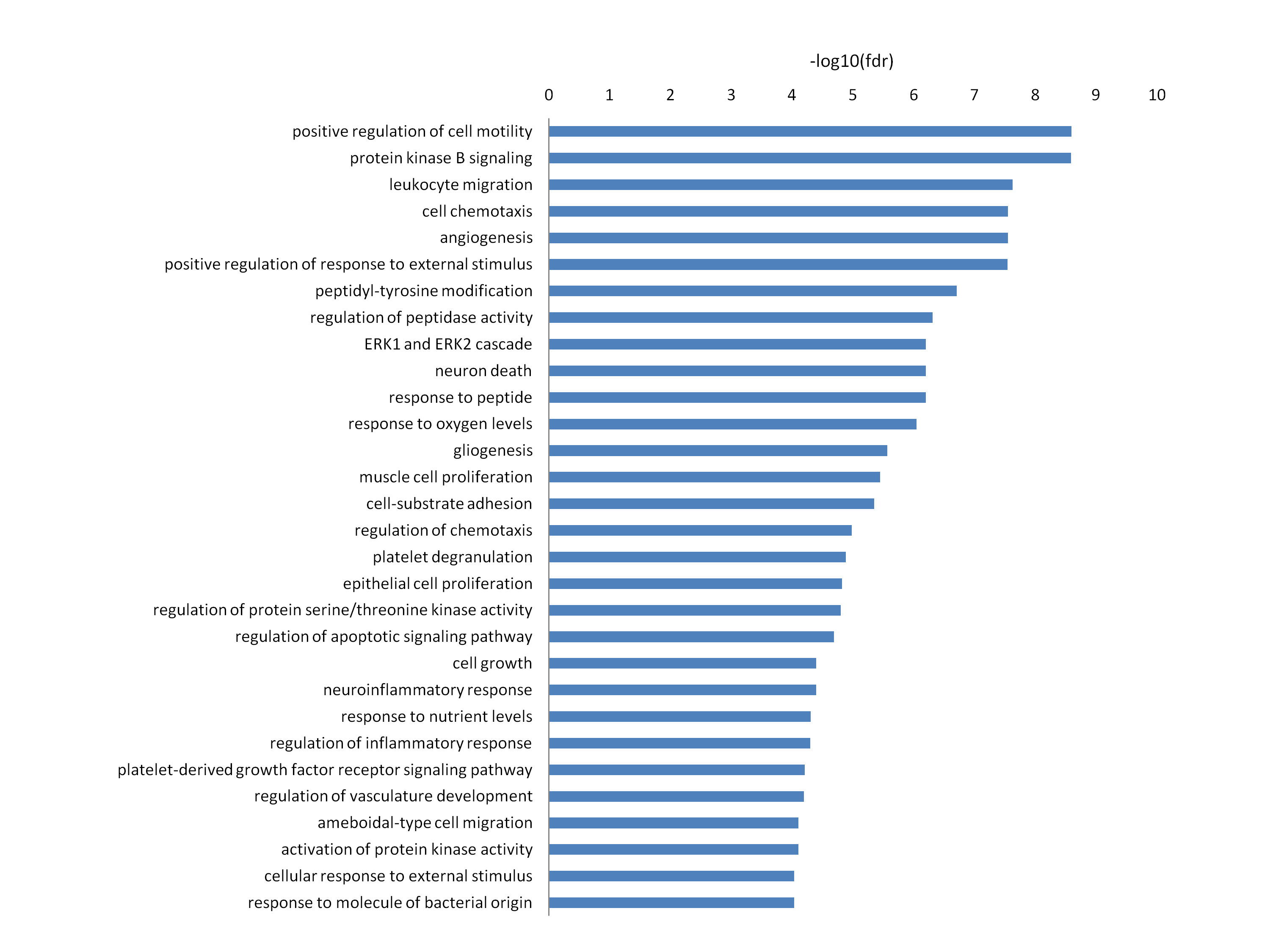

Supplement: Supplementary file 5 [file ACEL-19-e13256-s005.TIF]

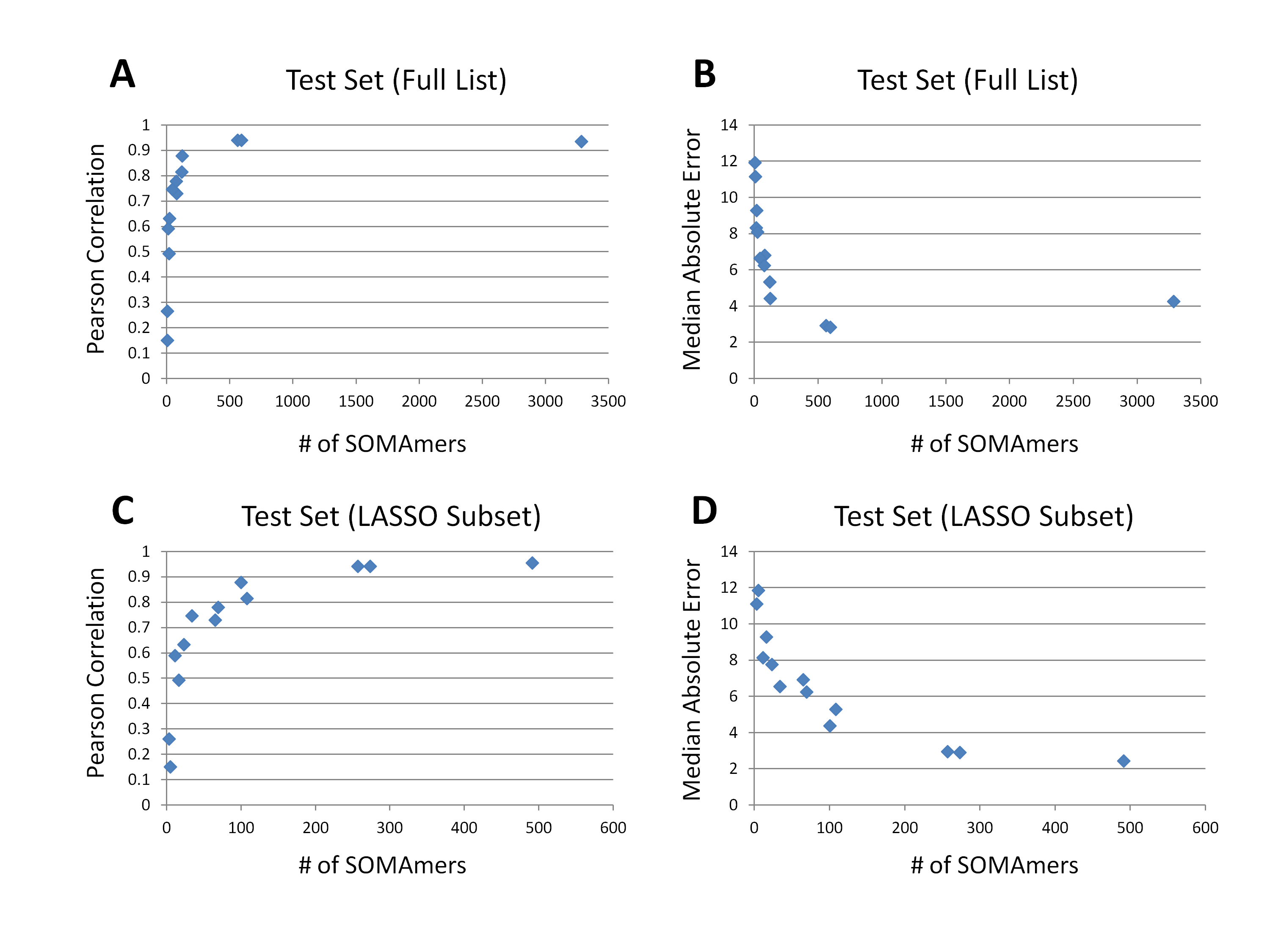

Supplement: Supplementary file 6 [file ACEL-19-e13256-s006.TIF]

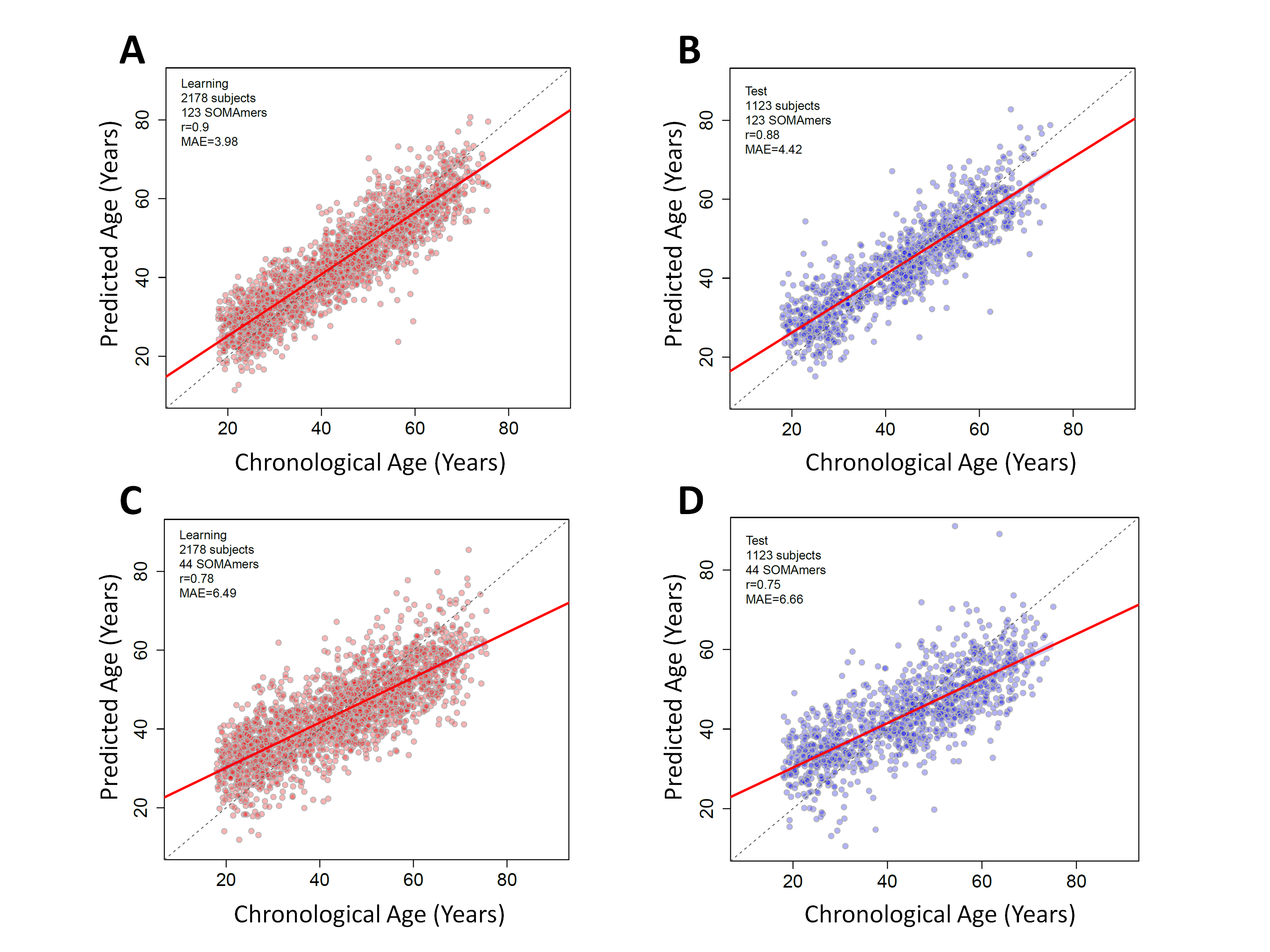

Supplement: Supplementary file 7 — Fig S7 [file ACEL-19-e13256-s007.TIF]

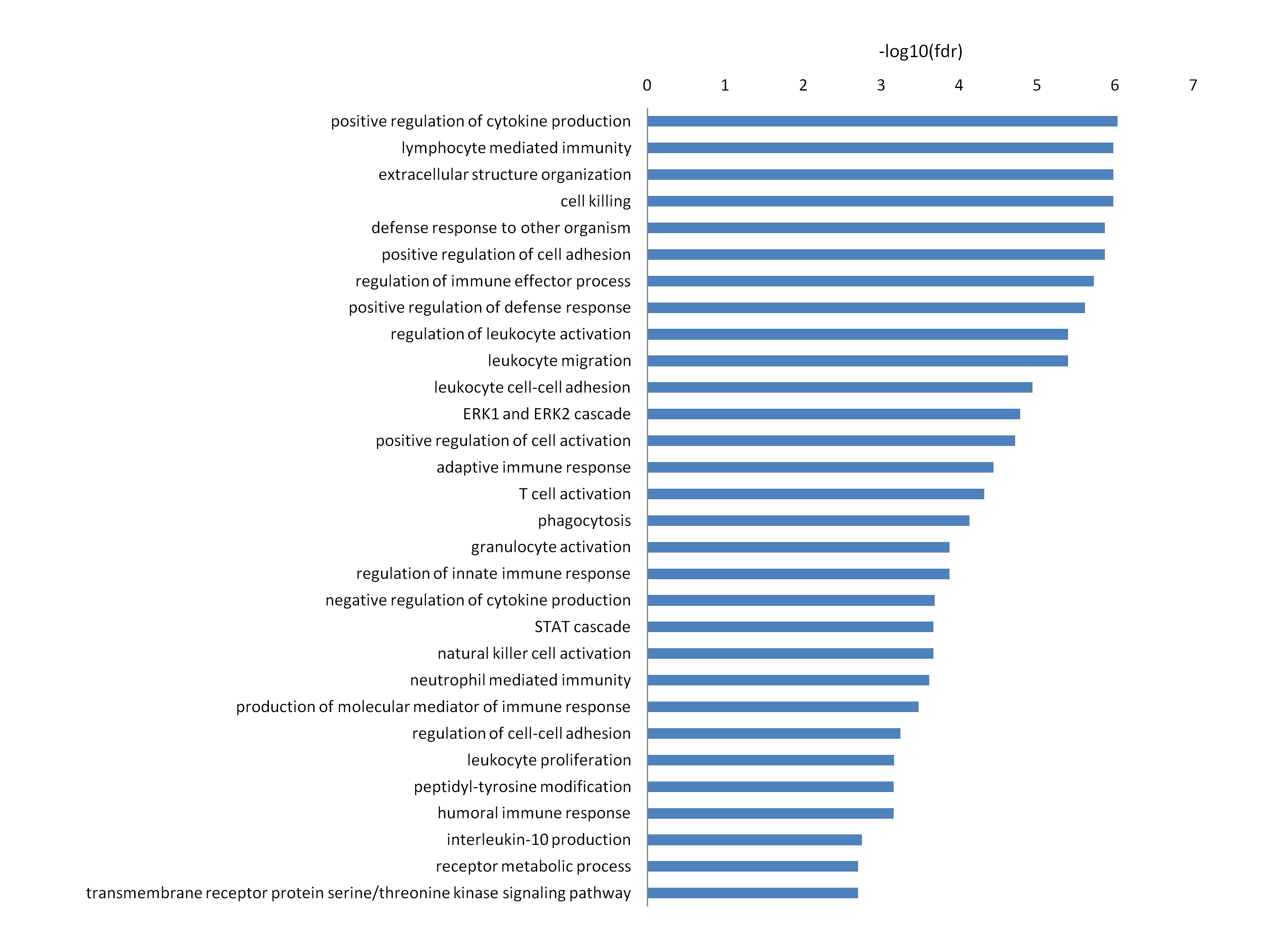

Supplement: Supplementary file 8 [file ACEL-19-e13256-s008.TIF]

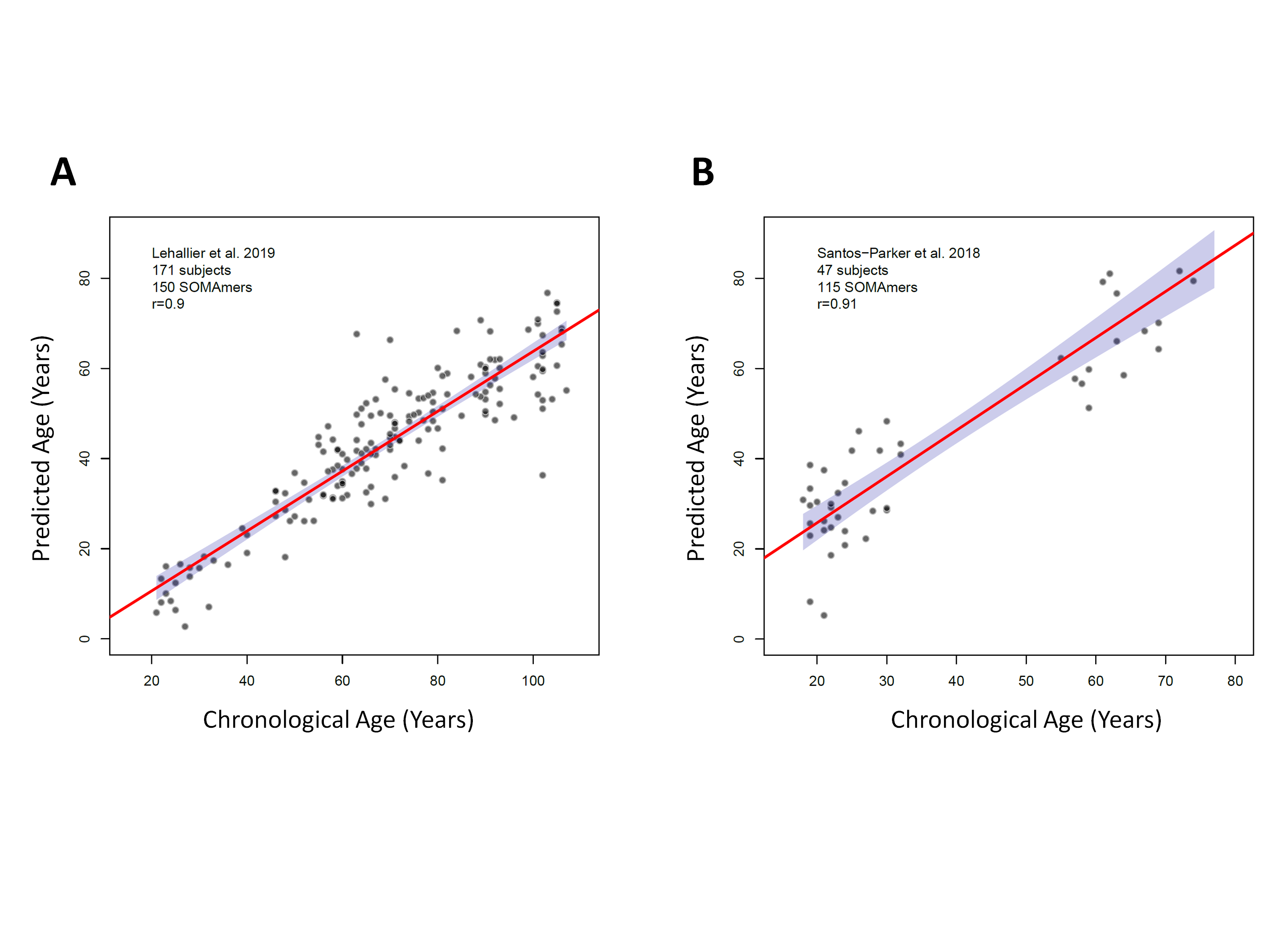

Supplement: Supplementary file 9 [file ACEL-19-e13256-s009.TIF]

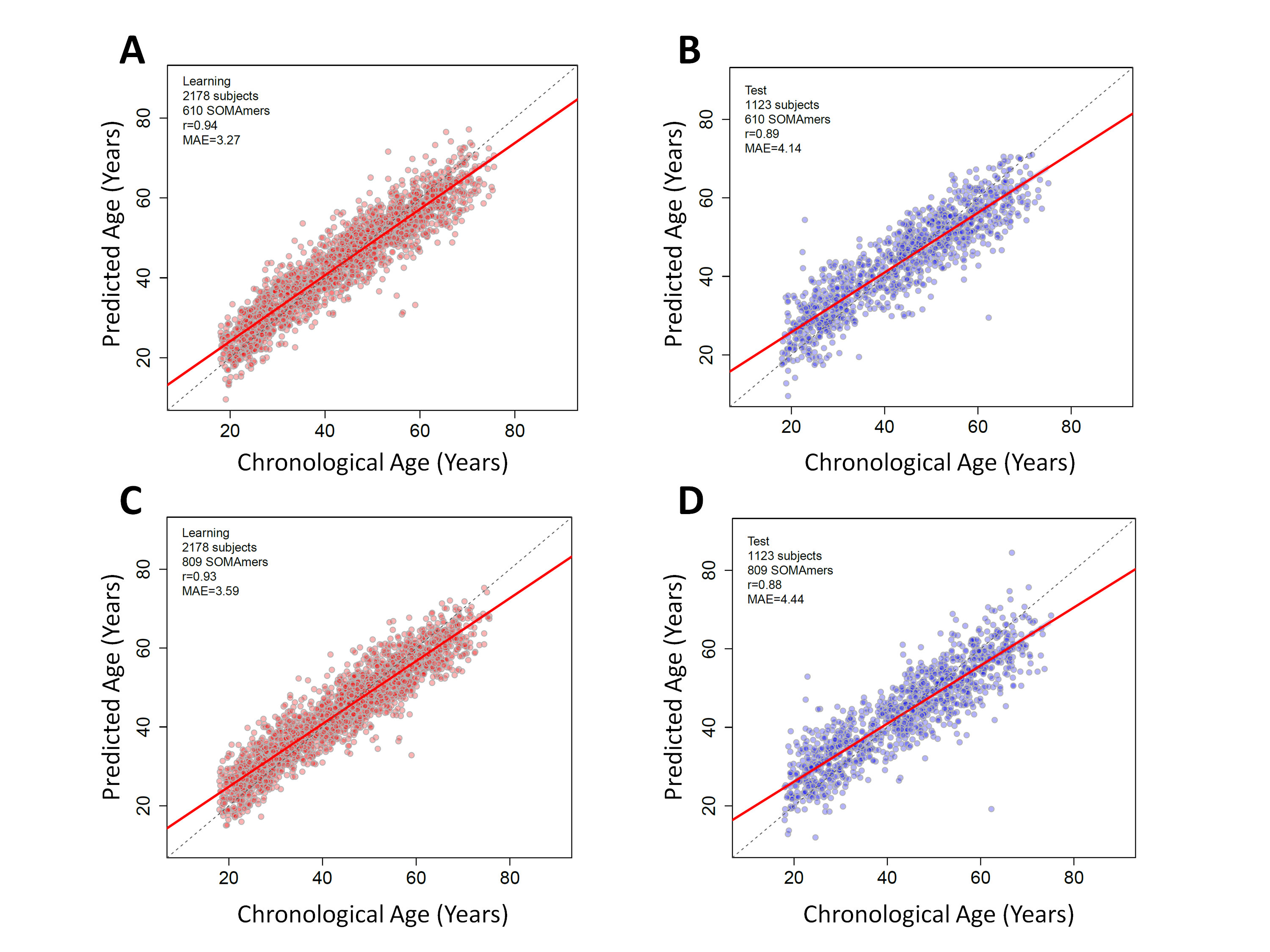

Supplement: Supplementary file 10 [file ACEL-19-e13256-s010.TIF]
