# Supplementary material for: Data mining of human plasma proteins generates a multitude of highly predictive aging clocks that reflect different aspects of aging
Source: Aging Cell. 2020 Oct 8;19(11):e13256. doi: 10.1111/acel.13256 (PMC7681068; doi:10.1111/acel.13256)
Supplement: Supplementary file 12 [file ACEL-19-e13256-s012.docx]

**SUPPLEMENTARY FIGURE LEGENDS**

**Supplementary Figure 1.** Expression changes in human plasma with age are shown for the following 20 proteins listed in Table 1: ADAMTS5 (A), BDNF (B), CCL11 (C), CGA.FSHB (D), FGA.FGB.FGG (E), IL15RA (F), IL6 (G), LIFR (H), LILRB2 (I), MMP12 (J), NAB1 (K), NTN1 (L), PAK4 (M), PLA2G2A (N), PLXNB2 (O), POMC (P), PRKAA1.PRKAB1.PRKAG1 (Q), RBM3 (R), SIRT5 (S), and UFM1 (T). RFU = relative fluorescence unit.

**Supplementary Figure 2.** Expression changes in human plasma with age are shown for the following nine proteins listed in Table 2: AKT2 (A), GDF11 (B), GDF15 (C), GHR (D), NAMPT (E), PAPPA (F), PLAU (G), PTEN (H), and SHC1 (I). RFU = relative fluorescence unit.

**Supplementary Figure 3.** A) All nine proteins identified to extend lifespan in normal mice or fish when manipulated were analyzed in the GLAD4U drug database. B) All 35 proteins identified to increase or decrease longevity in a normal vertebrate animal model were analyzed in the GLAD4U drug database. Enrichment results from the overrepresentation analyses are presented as -log10(fdr).

**Supplementary Figure 4**. A) An overrepresentation analysis in the Gene Ontology Biological Process database was performed for all 115 protein entries that significantly (q < 0.05) change their expression level with age in human plasma and have a negative age coefficient. B) An overrepresentation analysis was similarly performed for all 64 proteins identified to impact longevity when manipulated in normal, non-diseased animal models. All significant enrichment results are presented as –log10(fdr).

**Supplementary Figure 5.** An overrepresentation analysis in the Gene Ontology Biological Process database was performed for all 108 proteins capable of impacting longevity in any animal model (including genetically complex, stress, and disease models). The top 30 significant enrichment results are presented as –log10(fdr).

**Supplementary Figure 6.** For the proteomic aging clocks presented in Figure 3, the relationship between the number of SOMAmers included in the clock and Pearson correlation (A) or median absolute error (B) is shown. For the LASSO protein subsets identified for each of these clocks, the relationship between the number of SOMAmers included in the subset and Pearson correlation (C) or median absolute error (D) is shown.

**Supplementary Figure 7.** Predictive age vs. chronological age is plotted for proteins that regulate lifespan in any animal model for the learning (A) and test (B) sets. Similar plots are shown for proteins that regulate lifespan in a normal vertebrate model for the learning (C) and test (D) sets. MAE = median absolute error.

**Supplementary Figure 8.** An overrepresentation analysis in the Gene Ontology Biological Process database was performed for all 491 protein entries that make up the proteomic aging clock visualized in Figure 4. The top 30 significant enrichment results are presented as –log10(fdr).

**Supplementary Figure 9.** We tested our most predictive aging clock in two independent plasma proteomic datasets – one comprised of 171 individuals with an age range of 21-107 years (A) and one comprised of 47 individuals with an age range of 19-77 years (B). In the 171-person cohort, the Pearson correlation was 0.9 (A). In the 47-person cohort, the Pearson correlation was 0.91 (B).

**Supplementary Figure 10.** Predictive age vs. chronological age is plotted for proteins associated with “signal transduction” in the Reactome database for the learning (A) and test (B) sets. Similar plots are shown for proteins associated with “immune system” in the Reactome database for the learning (C) and test (D) sets. MAE = median absolute error.
